# Acinetobacter baumannii Can Survive with an Outer Membrane Lacking Lipooligosaccharide Due to Structural Support from Elongasome Peptidoglycan Synthesis

**DOI:** 10.1128/mBio.03099-21

**Published:** 2021-11-30

**Authors:** Brent W. Simpson, Marta Nieckarz, Victor Pinedo, Amanda B. McLean, Felipe Cava, M. Stephen Trent

**Affiliations:** a Department of Infectious Diseases, College of Veterinary Medicine, University of Georgia, Athens, Georgia, USA; b Department of Molecular Biology and Laboratory for Molecular Infection Medicine Sweden, Umeå Centre for Microbial Research, Umeå University, Umeå, Sweden; c Department of Microbiology, College of Art and Sciences, University of Georgia, Athens, Georgia, USA; KUMC

**Keywords:** ElsL, PBP1A, carboxypeptidase, cell envelope, lipopolysaccharide, outer membrane, peptidoglycan

## Abstract

Gram-negative bacteria resist external stresses due to cell envelope rigidity, which is provided by two membranes and a peptidoglycan layer. The outer membrane (OM) surface contains lipopolysaccharide (LPS; contains O-antigen) or lipooligosaccharide (LOS). LPS/LOS are essential in most Gram-negative bacteria and may contribute to cellular rigidity. Acinetobacter baumannii is a useful tool for testing these hypotheses as it can survive without LOS. Previously, our group found that strains with naturally high levels of penicillin binding protein 1A (PBP1A) could not become LOS deficient unless the gene encoding it was deleted, highlighting the relevance of peptidoglycan biosynthesis and suggesting that high PBP1A levels were toxic during LOS deficiency. Transposon sequencing and follow-up analysis found that axial peptidoglycan synthesis by the elongasome and a peptidoglycan recycling enzyme, ElsL, were vital in LOS-deficient cells. The toxicity of high PBP1A levels during LOS deficiency was clarified to be due to a negative impact on elongasome function. Our data suggest that during LOS deficiency, the strength of the peptidoglycan specifically imparted by elongasome synthesis becomes essential, supporting that the OM and peptidoglycan contribute to cell rigidity.

## INTRODUCTION

Gram-negative bacteria are particularly adept at surviving harsh environments due to their multilayered cell-envelope structure. The Gram-negative envelope consists of two lipid bilayers, namely, an inner and outer membrane (IM and OM, respectively), that encase the periplasmic aqueous compartment and a layer of peptidoglycan (1). As opposed to the symmetric glycerophospholipid (GPL) structure of the IM, the OM is asymmetric with GPLs in the inner leaflet and glycolipids, either lipopolysaccharide or lipooligosaccharide (LPS or LOS, respectively), on the cell surface. Gram-negative bacteria go to great extents to maintain OM asymmetry because it provides a unique permeability barrier (2, 3) and perhaps structural properties (4–7). To maintain the asymmetric OM, outer leaflet glycolipids are transported directly to their designated leaflet (1), and mislocalized GPLs are removed or degraded (2).

LPS/LOS provide critical permeability properties to the OM (3), but it has remained unclear why LPS/LOS are essential for most Gram-negative bacteria. Intriguingly, spirochetes, Gram-negative bacteria that often lack LPS/LOS, tend to have an obligate intracellular lifestyle, relying on the host cell environment. One of the few spirochetes that can grow in a wide variety of environmental conditions, namely, *Leptospira*, contains LPS (8), suggesting that LPS/LOS are needed for some key cellular roles other than OM permeability. Recently, biophysical experiments have revealed that LPS/LOS provide structural rigidity to the OM of Escherichia coli (4) which could be imparted by unique features of LPS/LOS. The membrane anchor of LPS/LOS, lipid A, is highly acylated (4 to 7 acyl chains depending on the organism) and typically negatively charged (1). The negative charges are coordinated by divalent cations, forming an electrostatic network at the surface of the cell (9). Finally, LPS/LOS contain sugar chains, core oligosaccharide, and O antigen (O antigen not present on LOS) that form a hydrophilic layer (1). Due to these properties, LPS/LOS molecules interact tightly and the OM has reduced fluidity compared with the IM (9–11), perhaps contributing to structural rigidity.

However, previous models have argued that peptidoglycan provides the principle mechanical properties. The peptidoglycan wall plays a major role in resisting osmotic and mechanical stresses and is critical for cell shape and rigidity (12–14). Peptidoglycan is a sturdy mesh built by glycosyltransferases that polymerize a polysaccharide backbone and transpeptidases that cross-link short peptide bridges between strands (15). Building and growing the structure require coordination between multienzymatic complexes of cytoskeletal proteins and synthetic and degradative enzymes. The elongasome and divisome are complexes that include shape, elongation, division, and sporulation (SEDS)-family glycosyltransferases, class B penicillin binding protein transpeptidases, and other subunits, which synthesize peptidoglycan along the axis/length of the cell and at septa, respectively (16–20). Finally, class A penicillin binding proteins contain both glycosyltransferase and transpeptidase activities and can synthesize peptidoglycan either axially or septally (15).

While LPS/LOS are essential in most Gram negative bacteria, a few species can survive with the complete loss of LPS/LOS, including Neisseria meningitidis, Moraxella catarrhalis, and Acinetobacter baumannii (21). The ability of A. baumannii to grow in the absence of LOS is of particular interest as this bacterium is classified by WHO as a priority pathogen for new antibiotic development (22). It causes an unparalleled number of hospital-acquired and multidrug-resistant infections (23). Furthermore, A. baumannii can become LOS deficient in response to selection with cationic antimicrobials (24), such as polymyxins, by inactivating early lipid A synthesis genes *lpxA*, *lpxC*, or *lpxD*. However, the clinical relevance of LOS-deficient, polymyxin-resistant A. baumannii is not known. LOS-deficient mutants have drastic growth defects in the lab and are susceptible to antibiotics that would normally be blocked by the OM (25), which would be expected to have severe fitness costs during pathogenesis.

Our group previously found that peptidoglycan remodeling may be critical for survival during LOS deficiency in A. baumannii. LOS-deficient mutants can be isolated only in strains with naturally low levels (undetectable by Western blot) or an absence of penicillin binding protein 1A (PBP1A), a class A penicillin binding protein (26). However, we previously could not identify any changes in the peptidoglycan composition that explained why PBP1A levels needed to be low (26). PBP1A was recently found to effect septa formation and colocalize with cell septa in A. baumannii (27). In addition, two l,d-transpeptidase domain-containing proteins become essential in A. baumannii lacking PBP1A that were also found to be critical during LOS deficiency (27). l,d-transpeptidases are periplasmic and form l,d-cross-links (3-3 linkage) in peptidoglycan, cross-link OM proteins to peptidoglycan, or modify peptidoglycan with d-amino acids (28–31). A. baumannii LdtJ was demonstrated to have a typical activity of forming l,d-cross-links (27). The second homolog, named both ElsL (32) and LdtK (27), was not required for l,d-cross-links (referred to as elongation and sulbactam sensitivity defects [ElsL] in this text for a more accurate nomenclature). Although no direct evidence was provided, ElsL was proposed by Kang et al. to cross-link OM proteins to peptidoglycan (27). Importantly, this study contradicted with its predicted cytoplasmic localization and did not consider that ElsL impacts the function of the elongasome (32).

We continued to study the critical role of LPS/LOS at the OM using LOS-deficient A. baumannii. Transposon (Tn) mutant libraries were generated in strains that contained or lacked LOS in the OM. Flanking regions of Tn insertions were sequenced (transposon insertion sequencing [Tn-seq]) (33, 34) to find genes that impacted fitness during LOS deficiency. Tn-seq identified that every member of the elongasome and ElsL were likely essential during LOS deficiency. Muropeptide and genetic analysis of mutants lacking ElsL suggested that this protein may instead be a cytoplasmic l,d-carboxypeptidase, involved in recycling of peptidoglycan fragments (15). ElsL and the elongasome were confirmed to be essential for the growth and viability of LOS-deficient mutants. We readdressed why high PBP1A levels were toxic to LOS-deficient mutants and found that high PBP1A levels in A. baumannii likely inhibit the activity of the elongasome. Altogether, we demonstrate that in the absence of LOS, the peptidoglycan rigidity specifically imparted by elongasome synthesis becomes essential, thus supporting the idea that both peptidoglycan and the asymmetrical OM support cellular integrity.

## RESULTS

### Peptidoglycan synthesis and remodeling pathways are critical in LOS-deficient A. baumannii.

To explore the critical role of LOS in the OM, we took a Tn-seq approach to identify genes that contribute to fitness in the absence of LOS. We built a saturated Tn library (∼136,000 mutants, 99% certainty of 2.5× coverage) (35) in wild-type (WT) strain 19606 (ATCC), which can become LOS deficient (26). However, Tn mutagenesis was ∼15-fold less efficient in 19606 LOS-deficient cells, likely due to the poor growth phenotype of this strain. LOS-deficient A. baumannii has growth defects that are suppressed by disrupting pathways that remove (Mla pathway) or degrade (PldA) GPLs from the outer leaflet of the OM (36, 37). These findings suggested that in the absence of LOS, GPLs were needed to fill the outer leaflet of the OM, and pathways that remove them were detrimental (36, 37). To overcome the reduced efficiency, a LOS-deficient 19606 Δ*mlaE* Δ*pldA* strain was tested. Tn mutagenesis was ∼5-fold more efficient, allowing us to build a saturated Tn library in an LOS-deficient strain (∼150,000 mutants, 99% certainty of 3× coverage). A saturated library was built in the isogenic LOS-containing 19606 Δ*mlaE* Δ*pldA* (∼157,000 mutants) strain for comparison.

**FIG 1:**
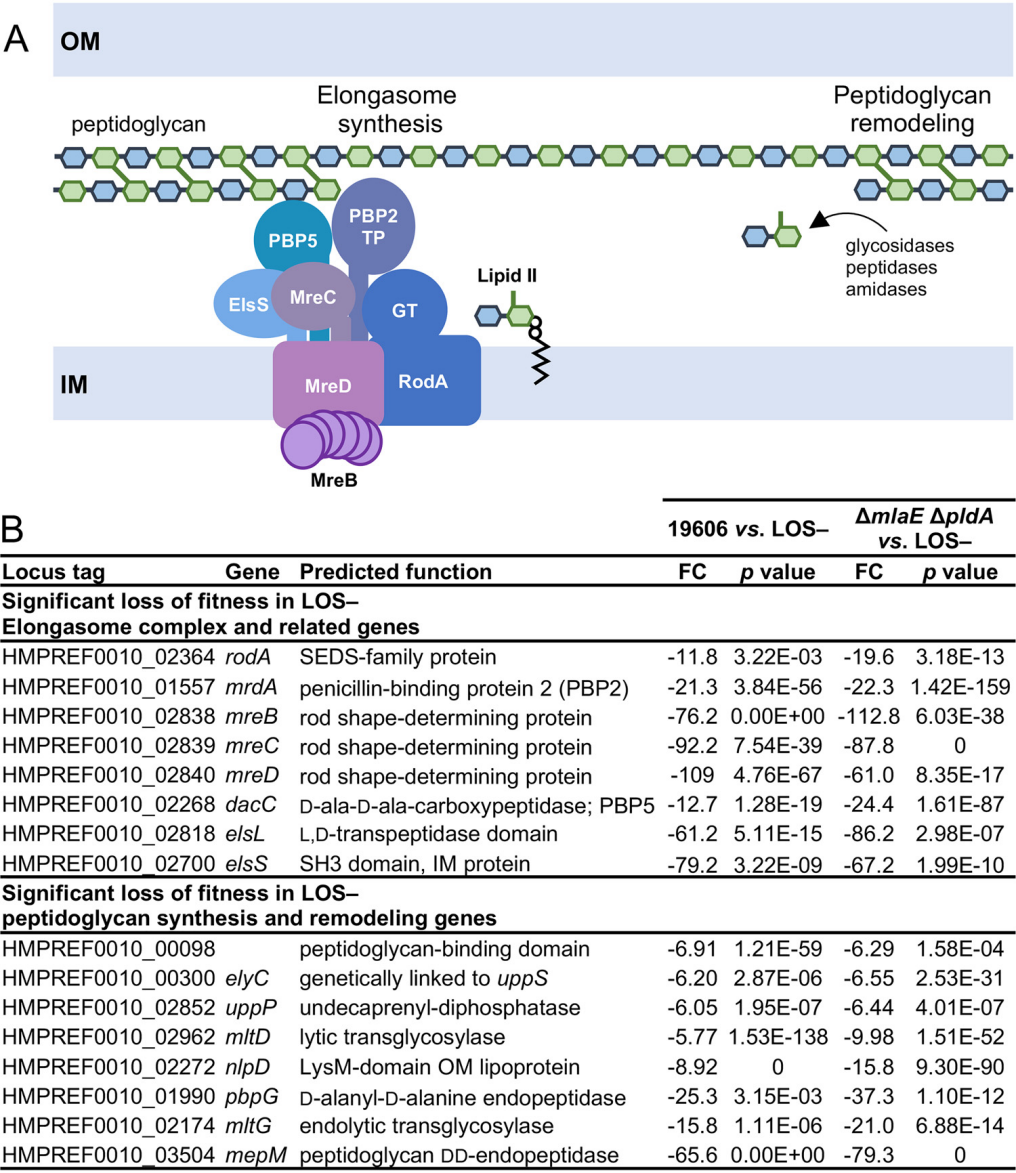
Peptidoglycan synthesis through the elongasome is critical for fitness of LOS-deficient A. baumannii. (A) Disruption of elongasome synthesis genes and peptidoglycan remodeling genes resulted in a loss of fitness during LOS deficiency. TP and GT indicate proteins with transpeptidase and glycosyltransferase activities, respectively, to incorporate lipid II precursors into the peptidoglycan layer. (B) Peptidoglycan synthesis and remodeling genes that when disrupted had a significant (≥5-fold) loss of fitness during LOS deficiency. FC indicates fold change.

In the LOS-deficient library, Tn insertions were underrepresented in 71 genes, classified as gene disruptions with a loss of fitness during LOS deficiency, and Tn insertions were overrepresented in 34 genes, classified as gene disruptions with increased fitness in LOS-deficient strains (see Fig. S1 in the supplemental material). In support of these latter candidate hits being biologically relevant, disruption of 16 genes involved in LOS synthesis or transport were essential in LOS-containing strains but were easy to disrupt in LOS-deficient strains. We chose to focus on peptidoglycan synthesis genes that impacted fitness in the absence of LOS since previous work had suggested some properties of peptidoglycan were critical for LOS deficiency. Every member of the elongasome (32) as well as genes associated with elongasome function were critical for fitness during LOS deficiency (Fig. 1 and Fig. S1). Unlike in many bacteria, the elongasome is nonessential in wild-type A. baumannii. In addition to conserved elongasome components, A. baumannii was found to contain the following three additional genes that impact elongasome function: *dacC* (HMPREF0010_RS15930, a d-ala-d-ala carboxypeptidase), *elsL* (HMPREF0010_RS18705, a cytoplasmic l,d-transpeptidase family protein), and *elsS* (HMPREF0010_RS18115, an SH3-domain, IM protein) (32). Disruption of these genes or elongasome-encoding genes resulted in a loss of rod shape and sensitivity to cell division inhibitors, like sulbactam (32). ElsS was demonstrated to interact with the elongasome subunit PBP2 by two-hybrid assays, perhaps acting as a scaffolding protein for the elongasome (32). However, the role of DacC and ElsL in elongasome function is still unclear. In addition to the elongasome, A. baumannii contains 3 other nonessential peptidoglycan synthesis enzymes, as follows: PBP1A, PBP1B, and MtgA (a monofunctional transglycosylase). We did not detect a significant impact on the fitness of LOS-deficient strains when these 3 genes were disrupted (Fig. S1), suggesting the elongasome was the only peptidoglycan synthesis pathway that was specifically required for growth of LOS-deficient cells.

10.1128/mBio.03099-21.1 FIG S1 Tn-seq during LOS deficiency. (A) Saturated Tn libraries were generated in an LOS-deficient strain and LOS-containing parent strains. Deep sequencing identified genes that when disrupted had a significant (≥5-fold) loss of fitness (left) or gain of fitness (right) during LOS deficiency. A summary of gene categories is provided for all hits. (B and C) Shows a graphical representation of read depth across representative genes with a scale on the left. Disruptions of representative genes, namely, *elsL* (B) and *rodA* (C), result in a significant loss of fitness in an LOS-deficient strain. Representative gene *mtgA* (E) has no significant difference in Tn insertions in an LOS-deficient strain. (D) Nonessential peptidoglycan synthesis genes that had no change in fitness (<5-fold) between LOS-containing and LOS-deficient strains. FC indicates fold change. Download FIG S1, PDF file, 0.4 MB. Copyright © 2021 Simpson et al. 2021 Simpson et al. https://creativecommons.org/licenses/by/4.0/ This content is distributed under the terms of the Creative Commons Attribution 4.0 International license.

### A. baumannii elongasome is critical for rod shape but does not majorly affect growth rate.

**FIG 2:**
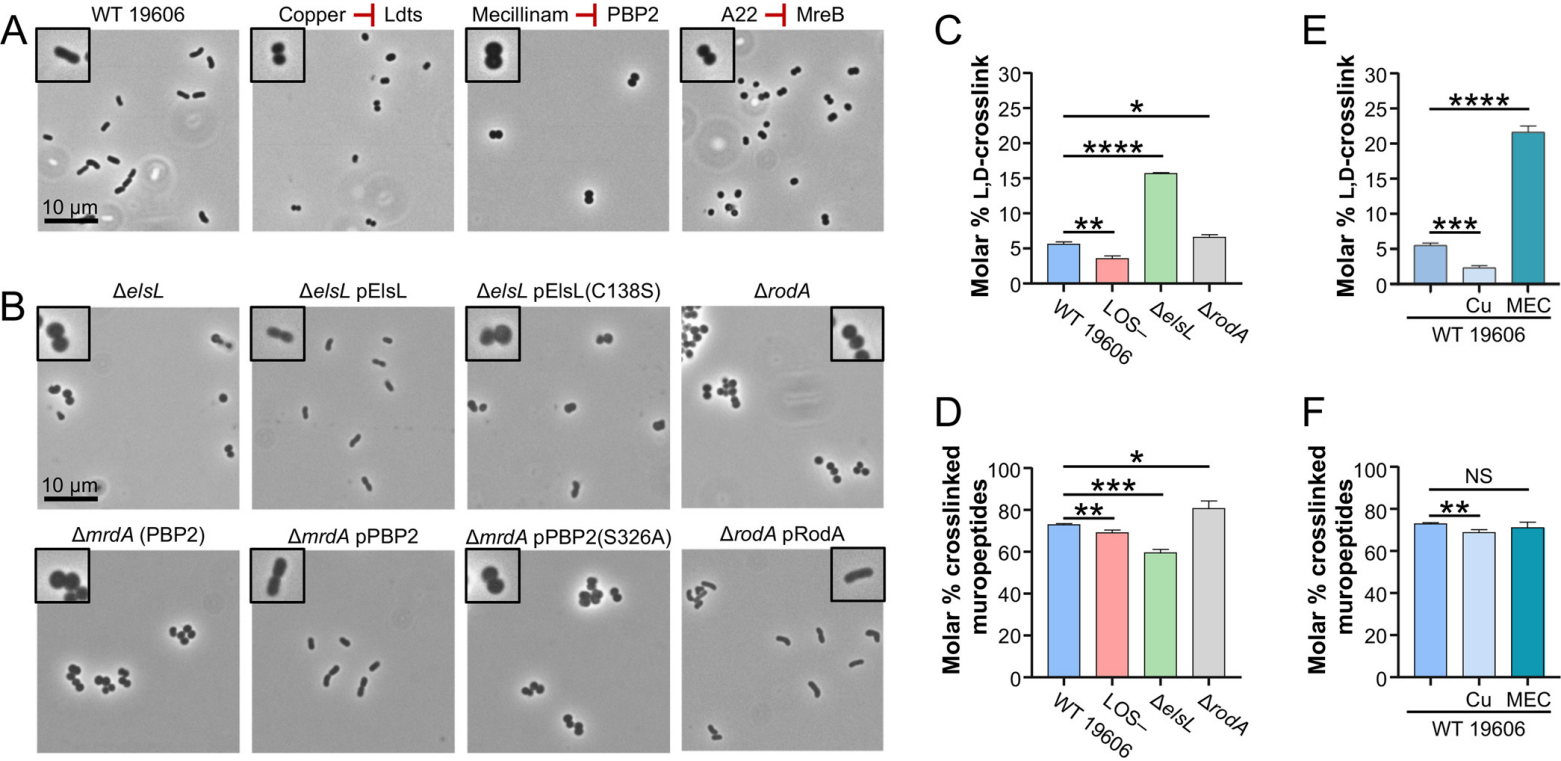
A. baumannii elongasome depends on the recycling subunit ElsL. (A) Inhibitors of ElsL [copper (II), 3.12 mM], PBP2 (mecillinam, 250 μg/mL), and MreB (A22, 15.6 μg/mL) disrupt activity of the A. baumannii 19606 elongasome and result in coccoid cells. (B) Disruption of ElsL and PBP2 (encoded by *mrdA*) by deletion or catalytic mutations disrupt activity of the elongasome, indicating that catalytic activity of both proteins is required for elongasome activity. Deletion and complementation of *rodA* were performed for comparison. IPTG was supplemented at 100 μM for pElsL, 25 μM for pPBP2, and 100 μM for pRodA. Phase-contrast microscopy was imaged at ×100 magnification, and all field of views were resized identically with a 10-μm scale bar on the first image of each panel. A single cell from each field of view is highlighted with a 2× magnified inset. (C to E) Assessment of l,d-cross-links (C and E) and overall cross-linking (D and F) detected by muropeptide analysis of 19606 mutants (C and D) or wild-type 19606 treated with chemical inhibitors (E and F), copper (II) (indicated as Cu) and mecillinam (indicated as MEC). Data represent mean values with the standard deviation from three independent cultures. Significance was calculated using Student’s unpaired *t* test. NS indicates not significant; *, *P *≤ 0.05; **, *P *≤ 0.01; ***, *P *≤ 0.001; ****, *P *≤ 0.0001.

We began by exploring the role of the elongasome in A. baumannii. Tn mutants that disrupt each gene of the elongasome (*dacC*, *elsL*, *elsS*, *mrdA* [encodes PBP2], *mreB*, *mreC*, *mreD*, and *rodA*) were cultivated from a Tn-mutant library in strain 5075 (38). Despite being fairly different pathogenic isolates, 19606 (urine isolate) and 5075 (osteomyelitis isolate) can both become LOS deficient (26), the 5075 library allowed a quick assessment of the role of many genes and testing of hypotheses in multiple A. baumannii strains to ensure effects were not strain specific. Tn mutants were evaluated for morphologic changes. As recently demonstrated in isolate 5075 (32), the disruption of genes associated with elongasome function resulted in a complete loss of the short rod shape of A. baumannii, with cells becoming shorter and rounder (see Fig. S2A in the supplemental material). We next built deletions of *elsL*, *mrdA*, and *rodA* in strain 19606. PBP2 and RodA were further investigated because they are the major peptidoglycan synthesis enzymes of the elongasome. ElsL, an unusual l,d-transpeptidase family protein, was investigated, as it was recently linked to the fitness of LOS-deficient A. baumannii (27). Δ*elsL*, Δ*mrdA*, and Δ*rodA* mutants similarly resulted in a loss of rod shape and shorter/rounder cells than those of the parent (Fig. 2).

10.1128/mBio.03099-21.2 FIG S2 All subunits of the elongasome are required for the rod shape. (A) Shows phase-contrast microscopy of 5075 Tn mutants that disrupt the elongasome. Microscopy was imaged at 100× magnification, and all fields of view were resized identically with a 10-μm scale bar on the first image of each panel. A single cell from the field of view is highlighted with a 2× magnified inset. (B) Shows doubling times of 5075 Tn mutants that disrupt the elongasome. (C) Doubling time of deletion mutants built in 19606. Above each, average doubling times and significant differences were assessed compared with the respective wild type from triplicate cultures as described in the Materials and Methods. *, *P *≤ 0.05; **, *P *≤ 0.01; ***, *P *≤ 0.001; ****, *P *≤ 0.0001. (D) Western blot of hemagglutinin (HA)-tagged ElsL and PBP2 variants expressed from the pMMB67EHtet plasmid. (E) Minimum inhibitory concentration of inhibitors that target peptidoglycan synthesis enzymes with standard deviations indicated from triplicate experiments. Rifampicin was included as a drug whose influx is blocked when LOS is present in the OM. The fold change of antibiotic sensitivity comparing 19606 and 19606 Δ*mlaE* Δ*pldA* demonstrates the effect on drug permeability due to a loss of OM asymmetry. A fold change of antibiotic sensitivity comparing the LOS-containing parent strain, 19606 Δ*mlaE* Δ*pldA*, to its LOS-deficient mutant, 19606 Δ*mlaE* Δ*pldA* LOS–, demonstrates hypersensitivities due to the loss of all LOS in the OM. Download FIG S2, PDF file, 0.6 MB. Copyright © 2021 Simpson et al. 2021 Simpson et al. https://creativecommons.org/licenses/by/4.0/ This content is distributed under the terms of the Creative Commons Attribution 4.0 International license.

Since LOS-deficient A. baumannii exhibits growth defects (25), it was possible that any gene disruption impacting growth could have a synthetic sick phenotype during LOS deficiency. We assessed if elongasome function affected growth rate in LOS-containing cells. Disruption of *elsL*, *dacC*, and *mrdA* had no significant difference in growth rate compared with the 5075 parent strain (24.1- to 24.8-min and 22.8-min doubling times, respectively). In addition, Tn mutants that disrupted *elsS*, *mreB*, *mreC*, *mreD*, and *rodA* had only slight, but significant, growth defects (25.9- to 28.9-min doubling times) (Fig. S2B). Similarly, in the 19606 background, the loss of *elsL*, *mrdA*, or *rodA* had only small or no significant difference in growth rate compared with the parent strain (Fig. S2C). Together, these results demonstrated that the elongasome function was critical for rod shape but did not have a large effect on growth rate in A. baumannii.

### The A. baumannii elongasome depends on ElsL.

Next, we explored whether the presence of each protein (protein-protein interactions) or their enzymatic activities were critical for elongasome function. Inhibitors have been described that individually target subunits of the elongasome, namely, mecillinam inhibits PBP2 (39, 40) and A22 inhibits MreB polymerization (41, 42). In addition, copper (II) can inhibit l,d-transpeptidase family proteins that utilize a catalytic cysteine (43). However, copper (II) inhibition is pleiotropic and also inhibits other processes that depend on cysteines, such as lipoprotein biogenesis and disulfide-bond formation (44, 45). We determined the MIC of each compound in wild-type 19606 (Fig. S2E) and assessed morphologic effects at subinhibitory concentrations. Treatment with copper (II), mecillinam, and A22 was found to cause a loss of rod shape and cell rounding (Fig. 2). Together, results of this chemical biology approach indicated that these compounds affected their expected targets and suggested that ElsL, PBP2, and MreB activities were required for elongasome function.

Catalytically deficient mutants of *elsL* and *mrdA* were generated based on studies of paralogs (30, 46) to further test these hypotheses. The 19606 Δ*elsL* and Δ*mrdA* mutants were transformed with plasmids expressing either the corresponding wild-type protein or catalytically deficient variants. Wild-type alleles of *elsL*, *mrdA*, and *rodA* complemented their respective deletions restoring rod shape (Fig. 2), whereas catalytic-deficient alleles, *elsL(C138S)* and *mrdA(S326A*), were unable to restore shape (Fig. 2). Catalytically dead variants were produced at similar levels as the respective wild-type proteins (Fig. S2D), indicating the variants did not drastically alter protein production or turnover. However, we cannot rule out whether the proteins were properly folded. These results support that the catalytic activity of each protein was required for elongasome function.

We wanted to determine how the unknown function protein ElsL affected the elongasome. The wild-type, an LOS-deficient mutant, Δ*elsL*, and Δ*rodA* strains were grown to mid-log phase, and the cellular peptidoglycan was isolated. Peptidoglycan was cleaved to muropeptides, and the composition was assessed by ultraperformance liquid chromatography (UPLC) (see Fig. S3 in the supplemental material). We also assessed muropeptides of wild-type cells treated with copper (II) or mecillinam for comparison (Fig. S3). In agreement with the finding that that ElsL was not a l,d-transpeptidase, deletion of the encoding gene did not decrease l,d-cross-links but instead caused a drastic increase (2.8-fold) in l,d-cross-links (Fig. 2C). Others also recently showed that deleting the *elsL* homolog in A. baumannii 17978 does not decrease l,d-cross-links and that the second l,d-transpeptidase family protein LdtJ was solely responsible for periplasmic l,d-cross-links (27). It was not surprising that the loss of ElsL did not fully mimic muropeptide changes during copper (II) inhibition (Fig. 2C to F), which would inhibit both ElsL and LdtJ.

10.1128/mBio.03099-21.3 FIG S3 Muropeptide analysis of A. baumannii 19606 strains. UPLC chromatograms of muropeptides from 19606 wild-type (A), 19606 LOS-deficient (B), 19606 Δ*elsL* (C), and 19606 Δ*rodA* (D) strains grown to mid-log phase in LB. UPLC of muropeptides from the wild-type strain treated with 3.12 mM of copper (II) (E; abbreviated as Cu) or treated with 250 μg/mL of mecillinam (F). Muropeptides are labeled as follows: M3, monomer disaccharide tripeptide; M4^G^, monomer disaccharide tetrapeptide with *Gly* at fourth position; M4, monomer disaccharide tetrapeptide; M5, monomer disaccharide pentapeptide; D34^G^, dimer disaccharide tritetrapeptide with one Gly at fourth position; D33, dimer disaccharide tritripeptide; D44^G^, dimer disaccharide tetratetrapeptide with one Gly at fourth position; D43, dimer disaccharide tetratripeptide; D34, dimer disaccharide tritetrapeptide; D44, dimer disaccharide tetrtetrapeptide; D45, dimer disaccharide tetrapentapeptide; M4N, anhydrous monomer disaccharide tetrapeptide; T433, trimer disaccharide tetratritripeptide; T443, trimer disaccharide tetratetratripeptide; T444, trimer disaccharide tetratetratetrapeptide; T445, trimer disaccharide tetratetr-pentapeptide; Tt4444, tetramer disaccharide tetratetratetratetrapeptide; D44N, anhydrous dimer disaccharide tetratetrapeptide; T444N, anhydrous trimer disaccharide tetratetratetrapeptide. Download FIG S3, PDF file, 0.3 MB. Copyright © 2021 Simpson et al. 2021 Simpson et al. https://creativecommons.org/licenses/by/4.0/ This content is distributed under the terms of the Creative Commons Attribution 4.0 International license.

Interestingly, the Δ*elsL* mutant most closely mimicked muropeptide changes during mecillinam inhibition (Fig. 2C versus 2E), causing increased l,d-cross-links. The muropeptide profile of the Δ*elsL* mutant and mecillinam treatment did not match the profile of the Δ*rodA* mutant. In the absence of RodA, other peptidoglycan synthesis processes, such as PBP1A, PBP1B, or the divisome, likely compensated resulting in increased peptidoglycan cross-links (Fig. 2C and D). Altogether, our data showed that ElsL affected the ratio of d,d- and l,d-cross-links in peptidoglycan, leading us to hypothesize that ElsL was an l,d-carboxypeptidase. These enzymes are involved in recycling peptidoglycan fragments in the cytoplasm and act on similar substrates as l,d-transpeptidases, tetra-peptides of peptidoglycan. Importantly, if ElsL functions as an l,d-carboxypeptidase, then the loss of this protein would result in aberrant peptidoglycan recycling (discussed below). Aberrant peptidoglycan recycling would inhibit PBP2 of the elongasome and could explain why *elsL* mutants phenotypically match elongasome mutants.

### Genetic evidence supports that ElsL is an l,d-carboxypeptidase.

**FIG 3:**
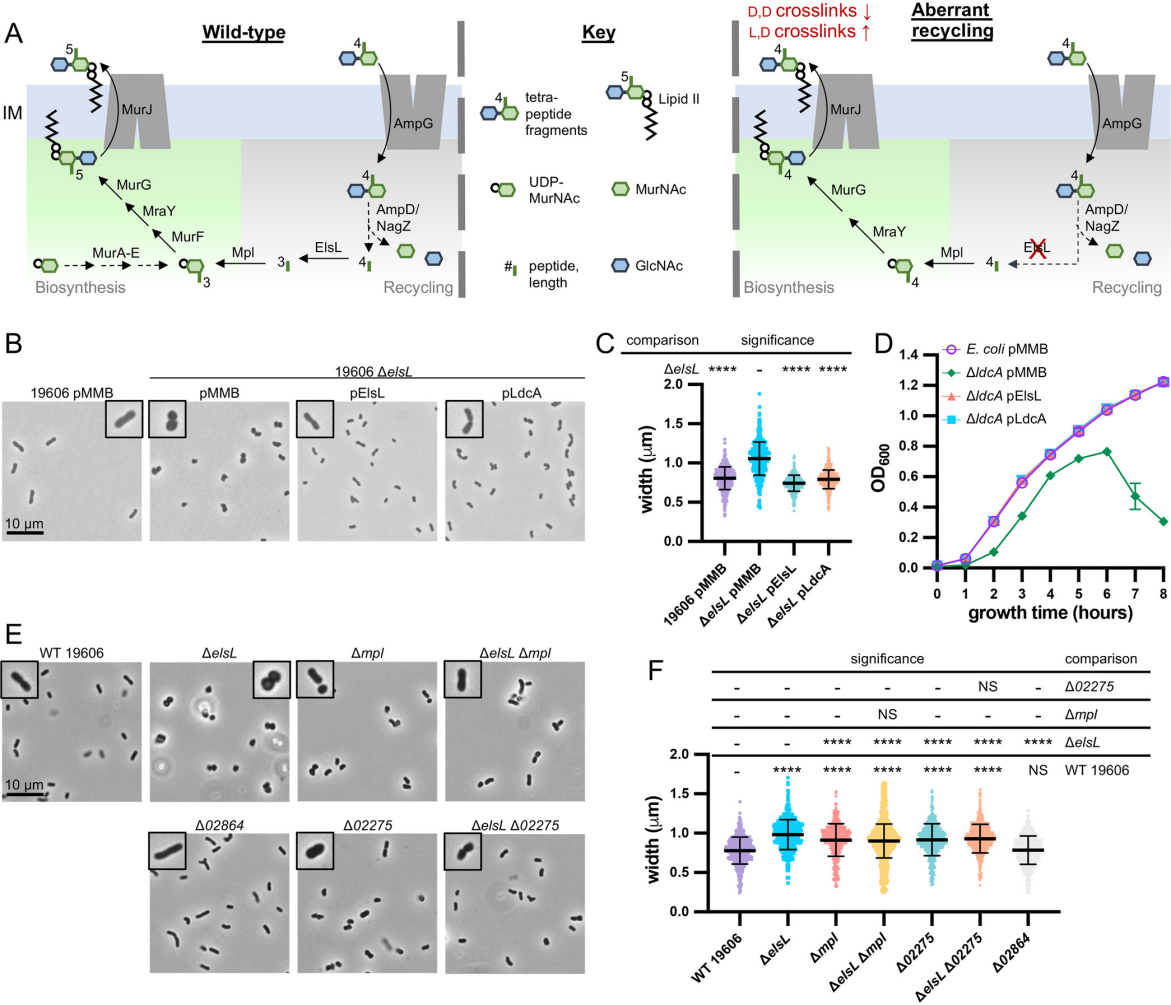
Genetic evidence supports that A. baumannii ElsL is an l,d-carboxypeptidase. (A) Depiction of peptidoglycan recycling pathway for tetrapeptide fragments released during peptidoglycan remodeling (left) and the aberrant recycling when an l,d-carboxypeptidase, like ElsL, is disrupted (right). Briefly, the permease AmpG imports tetrapeptide peptidoglycan fragments into the cytoplasm. Fragments are then degraded by a β-*N*-acetylglucosaminidase, NagZ; amidase, AmpD; and l,d-carboxypeptidase, ElsL, into individual sugars *N-*acetylglucosamine (GlcNAc; blue hexagon) and *N*-acetylmuramic acid (MurNAc; green hexagons), tripeptides, and d-alanine. Tripeptides can feed directly back into peptidoglycan synthesis through Mpl which ligates them to a fresh UDP-activated MurNAc, and they enter the biosynthesis pathway upstream of the d-ala-d-ala ligase MurF. In the absence of an l,d-carboxypeptidase (right), a tetrapeptide is instead released and ligated to UDP-MurNAc. MurF is bypassed because the fourth amino acid d-ala is already present and a tetrapeptide peptidoglycan precursor is produced. Tetrapeptide peptidoglycan precursors are unable to be utilized by d,d-transpeptidases but can be utilized by l,d-transpeptidases. (B and C) Phase-contrast microscopy of 19606 Δ*elsL* mutants with empty (pMMB) or complementation plasmids encoding ElsL from A. baumannii or the known l,d-carboxypeptidase LdcA from E. coli. (D) Averages of quadruplicate growth curves of E. coli Δ*ldcA* mutants with empty (pMMB) or complementation plasmids encoding ElsL from A. baumannii or LdcA, from E. coli. (E and F) Phase-contrast microscopy of 19606 mutants that disrupt peptidoglycan recycling genes *elsL* and *mpl* and two possible homologs of *ampG*, namely, *02275* and *02864*. Δ*mpl* and Δ*02275* mutants have the same morphological changes that are dominant to the cell rounding of the Δ*elsL* mutant, indicating their shared role in peptidoglycan recycling. Microscopy (B and E) imaged at ×100 magnification and all fields of view were resized identically with a 10-μm scale bar on the first image of each panel. A single cell from the field of view is highlighted with a 2× magnified inset. Cell measurements (C and F) were performed on ≥400 cells with MicrobeJ and assessed for significant differences as indicated in the Materials and Methods; NS indicates not significant; *, *P *≤ 0.05; **, *P *≤ 0.01; ***, *P *≤ 0.001; ****, *P *≤ 0.0001.

During peptidoglycan growth, disaccharide tetrapeptide fragments are released by endopeptidases and lytic transglycosylases (47). These fragments can then be taken up into the cytoplasm through the permease AmpG (48, 49) and reutilized via a recycling pathway (Fig. 3A). The β-*N*-acetylglucosaminidase NagZ cleaves off the *N-*acetylglucosamine (GlcNAc) (50, 51), and the amidase AmpD cleaves the bond between the peptide and *N*-acetylmuramic acid (MurNAc) (52, 53). Both sugars then can be reutilized in peptidoglycan precursor biosynthesis or enter sugar metabolism. Tetrapeptides are cleaved to tripeptides by l,d-carboxypeptidases (54). Mpl can ligate tripeptides to UDP-MurNAc (55) and feeds into peptidoglycan precursor biosynthesis just upstream of the addition of the two final d-ala-d-ala residues by MurF (56). A. baumannii contains homologs for every member of this recycling pathway but notably lacks a known l,d-carboxypeptidase.

Disruption of l,d-carboxypeptidases causes aberrant recycling of tetrapeptides (Fig. 3A) that results in decreased peptidoglycan synthesis and d,d-cross-linking, which is partially alleviated by increasing l,d-cross-links (57). We observed a similar profile for Δ*elsL* strains (Fig. 2C and D). In the absence of an l,d-carboxypeptidase, peptidoglycan recycling still occurs, resulting in the uptake and release of cytoplasmic tetrapeptides. Mpl ligases have broad substrate selectivity and will ligate tri-, tetra-, or pentapeptides to UDP-MurNAc (58). When tetrapeptides are ligated to UDP-MurNAc, the resulting precursor cannot be processed by MurF, as the fourth amino acid d-ala is already present. MurF is bypassed and a tetrapeptide peptidoglycan precursor is produced which can nonetheless be incorporated into the peptidoglycan polymer (57). However, tetrapeptide precursors cannot be utilized by d,d-transpeptidases like PBP2 of the elongasome, resulting in decreased d,d-cross-links.

To test if *elsL* encoded a novel l,d-carboxypeptidase, we attempted to complement the Δ*elsL* mutant with a known cytoplasmic l,d-carboxypeptidase, the E. coli LdcA. LdcA was ectopically expressed using a *p_tac_* promoter. Production of either ElsL or LdcA complemented cell shape changes of the *elsL* mutant and restored the rod shape (Fig. 3B). Since rounder cells have a greater cell width, measurements were performed (59). Δ*elsL* caused an increase of cell width that was complemented by either plasmid-carried *elsL* or *ldcA* (Fig. 3C). We performed the reciprocal experiment and tested if the A. baumannii *elsL* complemented phenotypes of an E. coli *ldcA* mutant. Disruption of E. coli *ldcA* results in stationary-phase lysis and altered cell shape, namely, shorter/rounder cells (54). The Δ*ldcA* allele from the Keio collection (60) was transduced using P1 phage into the E. coli W3110 wild-type strain and confirmed to cause stationary-phase lysis (Fig. 3D). Notably, suppressors of W3110 Δ*ldcA* occurred readily and the strain was not stable for repeated passages/storage. To overcome this issue, all other genetic manipulations were performed first, the Δ*ldcA* allele was transduced in last, and 4 transductants were assessed immediately after strain verification. Reproducibly, plasmid expression of either ElsL or LdcA complemented the stationary lysis phenotype as well as morphological changes of W3110 Δ*ldcA* (Fig. 3D; see Fig. S4A and B in the supplemental material).

10.1128/mBio.03099-21.4 FIG S4 ElsL belongs to a new family of l,d-carboxypeptidases. (A and B) A. baumannii ElsL complements cellular morphology changes of an E. coli Δ*ldcA* mutant. Phase-contrast microscopy (A) and cell measurements (B) of E. coli Δ*ldcA* mutants with empty (pMMB) or complementation plasmids encoding ElsL from A. baumannii or LdcA from E. coli. Microscopy was imaged at 100× magnification, and all fields of view were resized identically with a 10-μm scale bar on the first image of each panel. A single cell from the field of view is highlighted with a 2× magnified inset. Cell measurements were performed on ≥400 cells with MicrobeJ and assessed for significant differences as indicated in the Materials and Methods; *, *P* ≤ 0.05; **, *P* ≤ 0.01; ***, *P* ≤ 0.001; ****, *P* ≤ 0.0001. C) Substituted-cysteine accessibility method to assess localization of E. coli LdtA (top) and A. baumannii ElsL (bottom) in whole cells. Intact (N) cells were treated with cysteine-reactive compounds that either can (NEM) or cannot (MTSES) permeate through the inner membrane. The presence or absence of modifications was assessed after lysing cells and denaturing by the ability to block cysteine modification by a 2-kDa mal-PEG (Mal-PEG2k). (D) Maximum likelihood phylogeny of l,d-transpeptidase domain-containing proteins found in *Gammaproteobacteria*. The tree was tested with 100 bootstrap iterations. Branches with known function homologs are highlighted, and the homolog’s name, function, and domain architecture are shown. SP indicates the presence of a signal peptide predicted by SignalP5.0 (11). All other domains refer to Pfam (12) domains, as follows: YkuD=PF03734, l,d-transpeptidase catalytic domain; LysM=PF01476, LysM domain; Ldt_C=PF17969, l,d-transpeptidase C-terminal domain; PGbind1 = PF01471, putative peptidoglycan binding domain. Download FIG S4, PDF file, 0.6 MB. Copyright © 2021 Simpson et al. 2021 Simpson et al. https://creativecommons.org/licenses/by/4.0/ This content is distributed under the terms of the Creative Commons Attribution 4.0 International license.

Next, we tested if *elsL* had genetic interactions with other genes involved in recycling. Peptidoglycan fragment recycling becomes aberrant if the l,d-carboxypeptidase is disrupted, which depends both on the import of peptidoglycan fragments by AmpG and ligation of freed tetrapeptides to UDP-MurNAc by Mpl. In Vibrio cholerae, mutations disrupting *ampG* or *mpl* suppressed the toxic effect of an l,d-carboxypeptidase gene deletion by disrupting recycling upstream or downstream, respectively (57). A. baumannii has one *mpl* homolog (*HMPREF0010_03668*) and two *ampG* homologs, (*HMPREF0010_02275* and *HMPREF0010_02864*, notated as *02275* and *02864*, respectively). We deleted each gene in 19606 and assessed cellular morphology. Deleting *mpl* and *02275* resulted in the same change to cell shape; Δ*mpl* and Δ*02275* mutants were slightly wider rods than the wild-type parent but not as wide and not rounded like the Δ*elsL* mutant (Fig. 3E and F). In contrast, *02864* had no observable effect on cellular morphology (Fig. 3E and F), indicating that *02275* was likely the functional homolog of *ampG* in A. baumannii. We next deleted *mpl* and *02275* in the Δ*elsL* strain to test if they had synthetic relationships with *elsL*. The double mutants Δ*mpl* Δ*elsL* and Δ*02275* Δ*elsL* suppressed the cell rounding of the Δ*elsL* mutant and looked identical to the single Δ*mpl* and Δ*02275* mutants (Fig. 3E and F). These results indicated that Δ*mpl* and Δ*02275* were dominant to the Δ*elsL* mutation and that *elsL* was genetically linked to peptidoglycan recycling.

Although ElsL lacks any signal peptide, we also assessed if it was cytoplasmic or periplasmic. Since ElsL naturally contains a single cysteine residue, we used the substituted-cysteine accessibility method (SCAM) (61) to assess localization. SCAM probes the accessibility of cysteines to modification with an inner membrane-impermeable (methanethiosulfonate [MTSES]) or -permeable (*N*-ethylmaleimide [NEM]) compound. After whole cells were treated with each cysteine-reactive chemical, cells were lysed, proteins were denatured, and any remaining free cysteines were labeled with a 2-kDa maleimide-polyethylene glycol (Mal-PEG). NEM blocks both periplasmic and cytoplasmic cysteines preventing Mal-PEG labeling, whereas MTSES blocks only periplasmic cysteines from Mal-PEG labeling. ElsL expressed in whole cells of E. coli or A. baumannii was blocked only by NEM and not MTSES, indicating that ElsL was cytoplasmic as expected (Fig. S4C). As a control, E. coli LdtA, which naturally has a signal peptide, was confirmed to be periplasmic, blocked by MTSES and NEM, when expressed in E. coli or A. baumannii (Fig. S4C).

Multiple lines of genetic evidence support that ElsL belongs to a new class of l,d-carboxypeptidases within the l,d-transpeptidase domain family. To determine the taxonomy of this class of enzymes, we searched for l,d-transpeptidase genes within 10 major orders of *Gammaproteobacteria*. ElsL orthologs were identified in representative bacteria from 4 of the 10 orders searched, namely, *Pseudomonadales*, *Legionellales*, *Acidiferrobacterales*, and *Chromatiales* (Fig. S4D). All ElsL orthologs were predicted to be cytoplasmic (62), suggesting they also encode l,d-carboxypeptidases.

### Elongasome peptidoglycan synthesis is essential for cell integrity during LOS deficiency.

**FIG 4:**
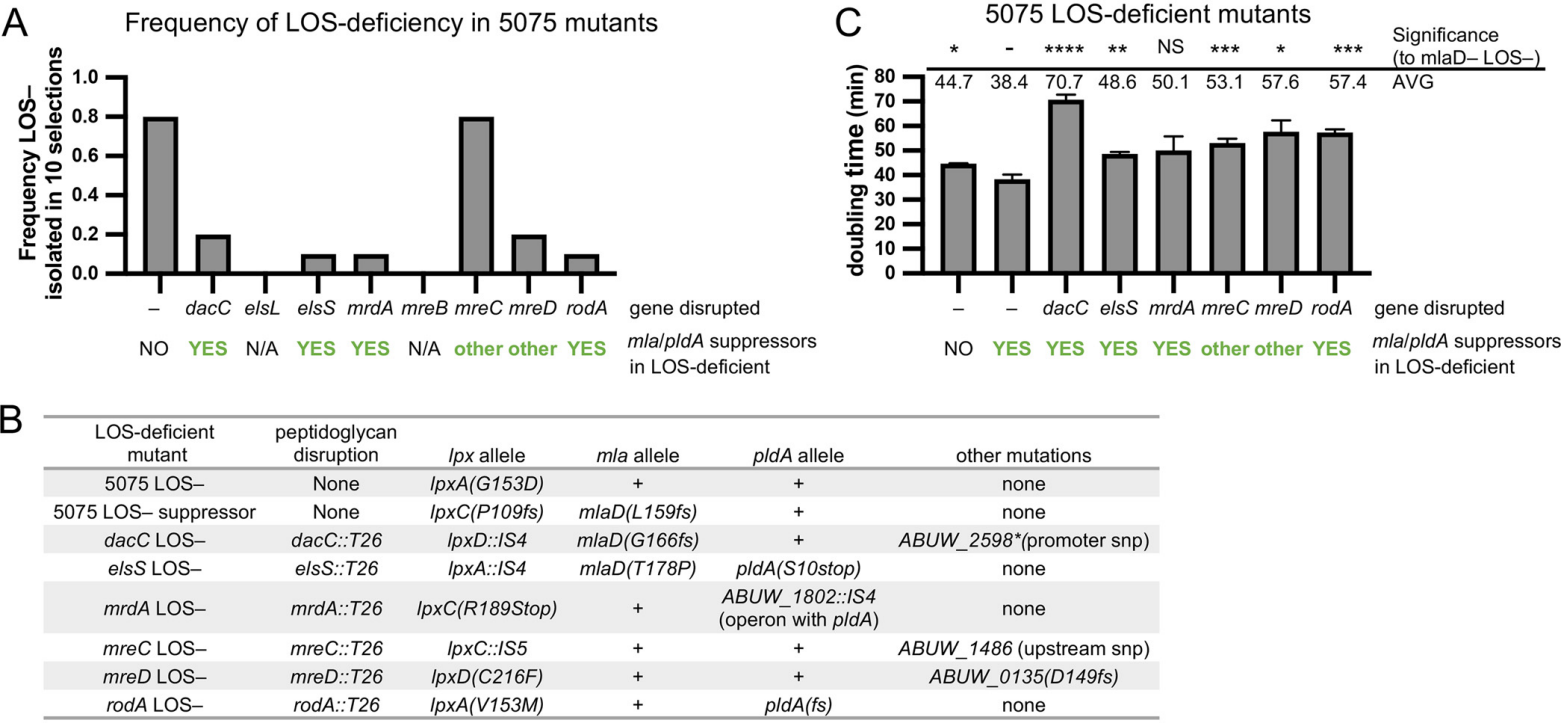
Elongasome function is critical for growth during LOS deficiency. (A) LOS-deficient mutants were rare in 5075 Tn mutants that disrupted the elongasome and quickly picked up the suppressor to stabilize the OM. Selections for LOS-deficient mutants were performed with 10 μg/ml of polymyxin B in 10 biological replicates per strain. (B) Tabular summary of mutations not found in the indicated strains isogenic parent shows that LOS/elongasome-deficient mutants quickly picked up suppressor mutations that predominantly disrupted *mla* genes, *pldA*, or both. (C) Doubling times from triplicate cultures of isolated LOS-deficient mutants. Since most LOS/elongasome-deficient mutants gained suppressor mutations in *mla* or *pldA* genes, growth rates were compared to a 5075 LOS-deficient suppressor that also inactivated *mlaD*. Above, average doubling times and significant differences, assessed as indicated in the Materials and Methods; *, *P *≤ 0.05; **, *P *≤ 0.01; ***, *P *≤ 0.001; ****, *P *≤ 0.0001.

To assess the role of the elongasome and peptidoglycan recycling during LOS deficiency, we again started by testing 5075 Tn mutants (38). LOS-deficient mutants can be selected on media containing polymyxin B, a drug that binds LOS, in order to disrupt the cell (24, 26). Polymyxin B selections were performed in 10 biological replicates to assess the frequency at which at least one LOS-deficient mutant could be isolated from a selection. We were never able to isolate LOS-deficient mutants in *elsL*- and *mreB*-disrupted mutants (Fig. 4A). In addition, LOS-deficient mutants were rarely isolated (≤2 out of 10 replicate selections) from 5075 Tn mutants with disruption of *dacC*, *mrdA*, *mreD*, or *rodA* (Fig. 4A; see Fig. S5A in the supplemental material). For the *mreC* Tn strain, we were able to isolate LOS-deficient mutants at a rate similar to that seen for the wild type (Fig. 4A and Fig. S5A).

10.1128/mBio.03099-21.5 FIG S5 LOS-deficient strains produce no LOS. (A) Gel electrophoresis and ProQ emerald stain of LOS isolated from 5075 Tn mutants that disrupt the elongasome and LOS-deficient mutants isolated from these strains. E. coli W3110 (labeled E. coli K-12) and Salmonella enterica serovar Typhimurium LT2 (labeled S. enterica) were used as controls for LOS and LPS staining, respectively. Since E. coli K-12 strains lack O-antigen, the LPS of these bacteria has a similar size to LOS. (B) Gel electrophoresis and ProQ emerald stain of LOS isolated from 19606 LOS-deficient strains 19606 LOS–, the Δ*mlaE* Δ*pldA* LOS-deficient strain used for building a Tn-mutant library (labeled Δ*mlaE* Δ*pldA* LOS– for Tn library), and the Δ*mlaE* Δ*pldA* Δ*lpxC* LOS-deficient strain used for experiments (labeled Δ*mlaE* Δ*pldA* LOS– for experiments). (C and D) Thin-layer chromatography (TLC) analysis of lipid A species (C) and glycerophospholipids (D) from 19606 LOS-deficient strains labeled with ^32^P_i_. Download FIG S5, PDF file, 0.6 MB. Copyright © 2021 Simpson et al. 2021 Simpson et al. https://creativecommons.org/licenses/by/4.0/ This content is distributed under the terms of the Creative Commons Attribution 4.0 International license.

When we attempted to grow the LOS/elongasome-deficient mutants under well-oxygenated rich medium conditions (5-ml cultures in LB broth with polymyxin B at 37°C with shaking) for characterization, it was apparent that many were unstable and quickly picked up suppressors. Several LOS/elongasome-deficient mutants had long lag times followed by rapid growth. Since suppressors had likely accrued in our LOS/elongasome-deficient mutants, we whole-genome sequenced a representative of each to identify possible suppressor mutations. Each strain was confirmed to have a mutation in early LOS synthesis genes (*lpxA*, *lpxC*, or *lpxD*) that conferred LOS deficiency (Fig. 4B and Fig. S5A). However, all LOS/elongasome-deficient mutants contained at least one suppressor mutation in *mlaD*, *pldA*, or other genes (Fig. 4B). As mentioned above, disruption of Mla and PldA, pathways that remove GPLs from the outer leaflet of the OM, generally suppress LOS-deficient mutants allowing them to grow faster (36).

We compared the growth of our LOS/elongasome-deficient mutants to that of both a 5075 LOS-deficient strain and a 5075 *mlaD-*LOS-deficient strain to control to some extent for suppressors that had accrued. We note that this comparison is not perfect, as there could be differences in how each set of suppressor mutations helped to improve growth. As previously reported (36), our 5075 *mlaD* LOS-deficient strain doubled more rapidly (∼38 min) then the otherwise wild-type LOS-deficient strain (∼45 min). Even with suppressors that had accrued, the LOS/elongasome-deficient strains had significantly altered doubling times, namely, 49 to 71 min (Fig. 4C). We did observe that some of the LOS+ parent strains also had slight but significant differences in doubling time from 5075 WT (Fig. S2B) (3.1 to 6.1 min), but the difference in doubling time of LOS-deficient mutants was clearly more dramatic (Fig. 4C) (10.2 to 32.2 min). The quick acquisition of suppressor mutations that still had reduced growth rates supported the idea that the elongasome was critical to support growth in the absence of LOS.

To characterize the role of the elongasome and ElsL during LOS deficiency in a more controlled manner, we determined the sensitivity of LOS-deficient mutants to elongasome inhibitors and copper (II). We switched back to testing 19606 strains so that we could compare a 19606 LOS-deficient strain and a 19606 Δ*mlaE* Δ*pldA* LOS-deficient mutant in order to distinguish how *mla* and *pldA* disruptions suppressed inactivation of the elongasome during LOS deficiency. Typically, polymyxin B selective pressure is maintained during experiments with LOS-deficient strains to prevent reversion. To avoid any possible synergy between polymyxin B and other drugs that could impact our results, we built a clean *lpxC* deletion in the 19606 Δ*mlaE* Δ*pldA* background, producing a stable LOS-deficient mutant incapable of reversion (Fig. S5B to D). We were unsuccessful at deleting *lpxC* in the 19606 strain, and so we screened for reversion before and after all experiments. Both LOS-deficient mutants showed hypersensitivity to elongasome inhibitors and copper (II), as follows: up to 16-fold sensitivity to mecillinam, 4-fold sensitivity to A22, and 2-fold sensitivity to copper (II) (Fig. S2E). While the effects of copper (II) inhibition are likely to be complicated by its pleiotropic effects, the sensitivity trend agreed with sensitivity to elongasome inhibitors. To assess whether this sensitivity to elongasome inhibitors could be linked to changes in antibiotic influx arising from alteration of the OM, we compared the sensitivity of the Δ*mlaD* Δ*pldA* double mutant to the 19606 wild type. Mecillinam, A22, and copper (II) are small polar compounds that should diffuse nonspecifically through porins and thus should not be impacted by LOS content of the OM, whereas hydrophobic drugs like rifampicin are normally blocked by LOS in the OM, but can partition through GPL bilayers (1). When GPLs were allowed to accumulate in the OM due to inactivation of Mla and PldA, strains became hypersensitive to rifampicin (19606 versus 19606 Δ*mlaE* Δ*pldA* in Fig. S2E). However, the 19606 Δ*mlaE* Δ*pldA* mutant had the same sensitivity to elongasome inhibitors as the 19606 wild-type strain (Fig. S2E), indicating that the influx of these inhibitors was not affected by changes to LOS content in the OM.

**FIG 5:**
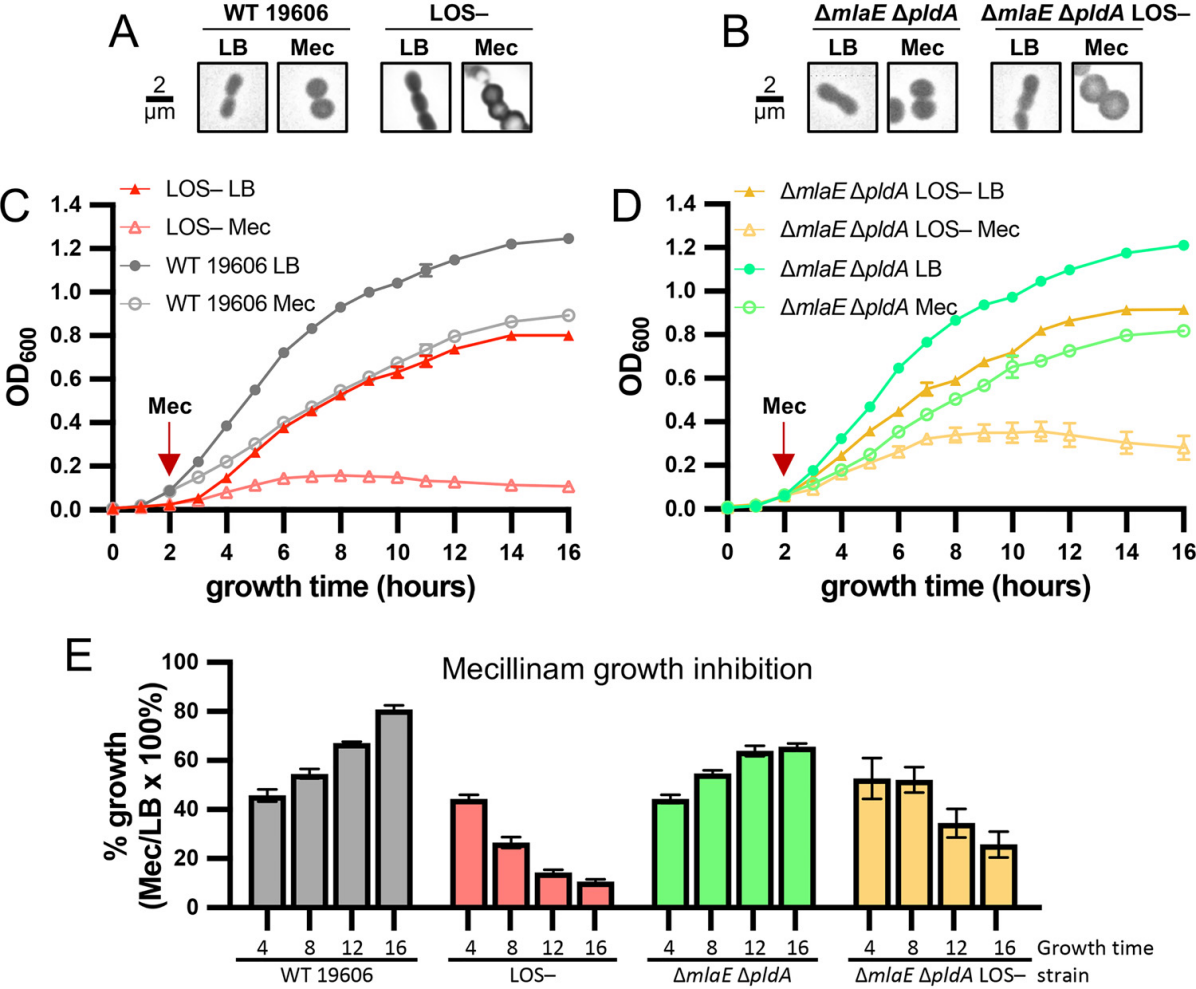
Elongasome function is essential for cell integrity during LOS deficiency. Chemical inhibition of PBP2 of the elongasome with 1,000 μg/mL of mecillinam of actively growing (added after 2 hours of growth) LOS-containing or LOS-deficient A. baumannii 19606 strains. (A and B) Two hours of exposure to mecillinam caused the same cell rounding for LOS-containing or LOS-deficient A. baumannii 19606 strains, suggesting elongasome activity was inhibited similarly. Phase-contrast microscopy imaged at ×100 magnification and all fields of view were resized as indicated for insets in Fig. 2. A 2-μm scale bar is provided. (C) Averages of triplicate growth curves of mecillinam-inhibited (open symbols) compared with LB-cultured (closed symbols) strains for the wild-type 19606 (circles) strain and 19606 LOS-deficient strain (triangles). Lysis of the 19606 LOS-deficient strain was evident after 6 hours of exposure to mecillinam (8 hours of growth). (D) Averages of triplicate growth curves of mecillinam-inhibited (open symbols) compared with LB-cultured (closed symbols) strains for the 19606 Δ*mlaE* Δ*pldA* (circles) and 19606 Δ*mlaE* Δ*pldA* LOS-deficient (triangles) strains. The 19606 Δ*mlaE* Δ*pldA* LOS-deficient strain grows somewhat with mecillinam inhibition, but lysis was evident after 8 hours of exposure (10 hours of growth). (E) Calculated percentage of growth of mecillinam--treated cultures compared with respective LB-untreated cultures.

Hypersensitivity of LOS-deficient mutants to select inhibitors supported the conclusion that the elongasome is critical during LOS deficiency. To determine if LOS-deficient cells were hypersensitive to elongasome inhibitors due to a cessation of growth or due to cell death, we grew strains to early log phase (2 hours in LB) and added levels of mecillinam that were inhibitory to LOS-deficient mutants. All strains had similar amounts of cell rounding at this concentration of mecillinam, and the elongasome was not essential to LOS-containing strains (Fig. 5A and B), indicating that this concentration of mecillinam was the appropriate level to cause significant elongasome inhibition in both LOS-containing (elongasome is not essential) and LOS-deficient strains (elongasome is essential). Mecillinam caused LOS-containing strains 19606 and 19606 Δ*mlaE* Δ*pldA* to slow growth within the first 2 hours of exposure (∼45% of growth in LB), but the growth of mecillinam-treated cultures recovered with ∼65% to 80% of the maximum optical density (OD) of cultures without antibiotic (Fig. 5). The 19606 LOS-deficient strain had the same ∼45% growth inhibition within a short 2-hour exposure to mecillinam, but the strain continued to slow growth and had lysis with longer exposure to mecillinam (Fig. 5; see Fig. S6 in the supplemental material), as assessed with microtiter plate live-dead assays. The 19606 Δ*mlaE* Δ*pldA* LOS-deficient strain also had an initial growth inhibition of ∼50% within 2 hours of treatment with mecillinam but continued to grow at this lower rate for several hours (Fig. 5D and E). However, the 19606 Δ*mlaE* Δ*pldA* LOS-deficient strain still had lysis with prolonged exposure (Fig. 5 and Fig. S6). Together, these results indicated that the elongasome was essential for growth (reduced growth capacity) and cellular integrity (lysis) of LOS-deficient mutants. Furthermore, although Mla- and PldA-inactivating suppressors can help to rescue the slow growth of LOS/elongasome-deficient cells, they cannot restore integrity to these cells.

10.1128/mBio.03099-21.6 FIG S6 Disruption of the elongasome during LOS deficiency results in cell lysis. Chemical inhibition of PBP2 of the elongasome with 1,000 μg/mL of mecillinam of actively growing (added after 2 hours of growth) LOS-containing or LOS-deficient A. baumannii 19606 strains. (A) Averages of triplicate growth curves of mecillinam-inhibited (open symbols) compared with those of LB-cultured (closed symbols) strains for the wild-type 19606 (circles) and 19606 LOS-deficient (triangles) strains. (B) Averages of triplicate growth curves of mecillinam-inhibited (open symbols) compared with those of LB-cultured (closed symbols) strains for the 19606 Δ*mlaE* Δ*pldA* (circles) and 19606 Δ*mlaE* Δ*pldA* LOS-deficient (triangles) strains. (C) Microtiter live-dead assays to assess the amount of cell lysis with prolonged exposure to mecillinam. The % lysed cells were calculated from a standard curve of intact and lysed cells of the wild-type 19606 strain as described in the Materials and Methods. (D to F) Increasing external osmotic pressure, 5% sucrose (suc), protects mecillinam-inhibited LOS-deficient cells from lysing. Mecillinam-inhibited (open symbols) cultures compared with LB cultures (closed symbols) with and without 5% sucrose of 19606 Δ*mlaE* Δ*pldA* (D) and 19606 Δ*mlaE* Δ*pldA* LOS-deficient (E) strains. (F) Calculated decline in OD_600_ during mecillinam treatment. Download FIG S6, PDF file, 0.3 MB. Copyright © 2021 Simpson et al. 2021 Simpson et al. https://creativecommons.org/licenses/by/4.0/ This content is distributed under the terms of the Creative Commons Attribution 4.0 International license.

Because peptidoglycan is known to provide cellular rigidity (12–14) and LPS/LOS have recently also been proposed to impart rigidity to the OM (4), elongasome synthesis of peptidoglycan could be imparting critical rigidity to compensate for the loss of rigidity to the OM in LOS-deficient cells. If LOS-deficient cells were lysing during elongasome inhibition due to the loss of critical cellular rigidity, then this lysis should be prevented by adding external osmolytes, such as sucrose. Mecillinam inhibition was reassessed with excess sucrose (5% [wt/vol]; ∼146 mM), which prevented lysis for the 19606 Δ*mlaE* Δ*pldA* LOS-deficient strain (Fig. S6D to F). These findings support the hypothesis that lysis of LOS-deficient bacteria during mecillinam inhibition occurs due to a reduction in cellular rigidity that was no longer sufficient to resist internal turgor pressure.

### High PBP1A levels may inhibit the elongasome to prevent LOS deficiency.

PBP1A protein levels have also been demonstrated to impact whether or not A. baumannii can become LOS deficient (26). A. baumannii strains with naturally high PBP1A levels, like 17978, are unable to become LOS deficient but can become LOS deficient if the encoding gene *ponA* is disrupted (26). Recently, PBP1A of A. baumannii was demonstrated to localize to the cell septa and affected the number of septa that were formed, indicating that it may have a role in coordinating cell division (27). It was hypothesized that an increased number of cell septa when PBP1A levels were low somehow stabilizes LOS-deficient cells (27). Increased cell septa were proposed to either slow growth to match a lower rate of building the OM or to increase the number of cell division complexes per cell that physically connects the IM and OM (27). However, there is precedent that PBP1A could instead affect the elongasome either through competition for peptidoglycan precursors or direct interaction. In Bacillus subtilis, overexpression of MreBCD caused cells to become longer and thinner, while overexpression of PBP1 (homolog of PBP1A) caused cells to become shorter and wider, suggesting that competing activities between the two complexes impact cell shape (63). In addition, PBP1A was demonstrated to interact with elongasome subunits in both A. baumannii and E. coli (32, 64).

**FIG 6:**
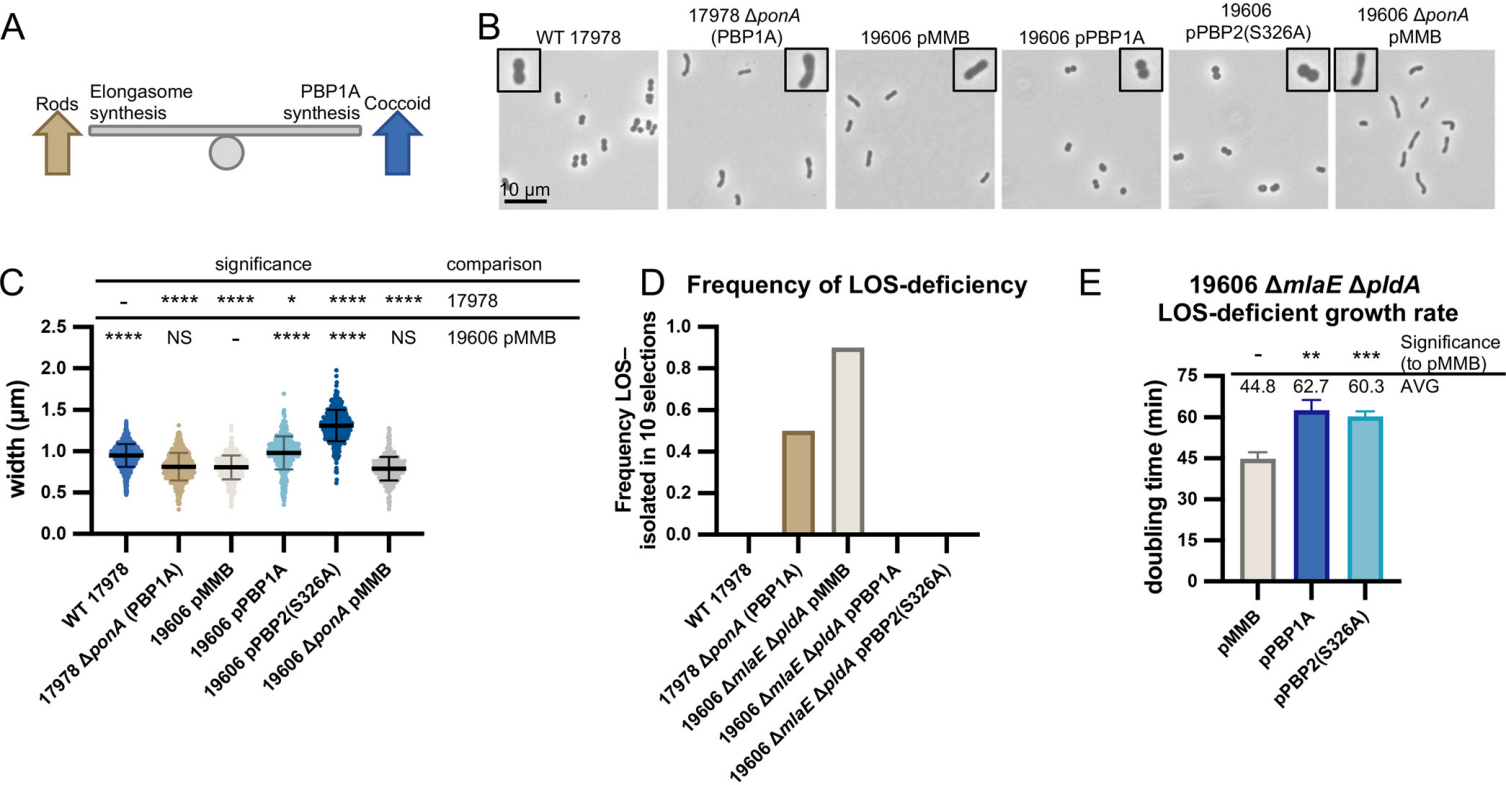
High PBP1A levels inhibit the elongasome which is toxic to LOS-deficient A. baumannii. (A) Observed balance between elongasome and PBP1A peptidoglycan synthesis and effects on cell shape. High elongasome synthesis results in longer rod-shaped cells, while high PBP1A synthesis results in shorter and coccoid cells. (B and C) Phase-contrast microscopy and cell measurements of 17978 and 19606 strains with changes in PBP1A protein levels. Overexpression of PBP1A and dominant-negative elongasome subunit PBP2(S326A) resulted in the same coccoid phenotype, suggesting high PBP1A levels inhibit the elongasome (25 μM IPTG). Microscopy imaged at ×100 magnification and all field of views were resized identically with a 10-μm scale bar on the first image of each panel. A single cell from the field of view is highlighted with a 2× magnified inset. Cell measurements were performed on ≥400 cells with MicrobeJ and assessed for significant differences as indicated in the Materials and Methods. (D) High PBP1A expression levels that caused coccoid morphology correlated with an inability to isolate LOS-deficient mutants. Shows the frequency of LOS-deficient mutants out of 10 replicate selections. (E) Inhibition of the elongasome when PBP1A or PBP2(S326A) were overexpressed in a stable 19606 Δ*mlaE* Δ*pldA* LOS-deficient strain resulted in severe growth defects. Above, average doubling times and significant differences, assessed from triplicate cultures as described in the Materials and Methods. NS indicates not significant; *, *P *≤ 0.05; **, *P *≤ 0.01; ***, *P *≤ 0.001; ****, *P *≤ 0.0001.

To determine if PBP1A levels impacted the elongasome, we compared the cell shape of 17978, which naturally has high PBP1A levels, and 19606, which naturally has low PBP1A levels that were previously undetectable by Western blot (Fig. 6) (26). Confirming results from other groups (27), we found that the wild-type 17978 cells were shorter and wider than wild-type 19606 cells. We next tested if we could swap these traits by altering PBP1A levels. A 17978 Δ*ponA* strain and 19606 strain with PBP1A expressed from an isopropyl-β-d-thiogalactopyranoside (IPTG)-inducible plasmid were assessed for cell shape. Notably, deletion of *ponA* in 17978 caused cells to become longer and thinner, mimicking the shape of 19606 wild-type strain cells (Fig. 6B and C). The opposite effect was observed when PBP1A was overexpressed in 19606 (19606 pPonA-HA), with cells becoming rounder mimicking cells of wild-type 17978 (Fig. 6B and C).

We noted that 17978 wild-type cells and 19606 cells overexpressing PBP1A were more coccoid-like elongasome mutants, supporting the idea that the elongasome activity could be inhibited by high PBP1A levels. Alternatively, high PBP1A activity could outcompete the elongasome for the limited pool of lipid II precursors. We reasoned that overexpressing a catalytic deficient subunit of the elongasome should also inhibit elongasome activity because it would titrate away interacting partners. Overexpression of the PBP2(S326A) catalytically inactive variant in 19606 caused a similar cell rounding to when PBP1A levels were increased (Fig. 6B and C). Together, these results supported the conclusion that PBP1A levels affect elongasome activity.

To test if these manipulations of PBP1A and elongasome activities correlated with the ability to become LOS deficient, we performed LOS-deficient selections. As observed previously (26), 17978 wild-type strains could not become LOS deficient, but LOS-deficient mutants could be selected for in the 17978 Δ*ponA* strain (Fig. 6D). Selections for LOS-deficient mutants while PBP1A levels were increased were performed in the 19606 Δ*mlaE* Δ*pldA* background to control for suppressors that can arise during selections. Overexpression of PBP1A or the dominant-negative PBP2 variant also caused coccoid morphology in the 19606 Δ*mlaE* Δ*pldA* background (see Fig. S7 in the supplemental material). The 19606 Δ*mlaE* Δ*pldA* strain with an empty plasmid could become LOS deficient, but overexpression of PBP1A or the dominant-negative PBP2 variant at levels that caused coccoid morphology prevented isolation of LOS-deficient mutants (Fig. 6D). These results indicated that high PBP1A levels or inhibition of the elongasome prevented the selection of LOS-deficient mutants. We also assessed the effect of overexpression of PBP1A and inhibition of the elongasome in cells that were already LOS deficient. Plasmids encoding PBP1A or the dominant-negative PBP2 variant were transformed into the stable 19606 Δ*mlaE* Δ*pldA* LOS-deficient strain under repressive conditions to prevent expression of the potentially toxic proteins. Upon induction of either protein at levels that cause coccoid morphology, there was a significant and drastic decrease in growth rate (∼60- to 63-min doubling time compared with ∼45 min for the empty plasmid control strain) (Fig. 6E). Altogether, our results show that overexpression of PBP1A or inhibition of the elongasome with a dominant-negative mutant had the same inhibitory effect on LOS-deficient mutants.

10.1128/mBio.03099-21.7 FIG S7 Overexpression of PBP1A inhibits the elongasome but does not affect the growth rate of LOS-containing A. baumannii. (A) Phase-contrast microscopy of 19606 Δ*mlaE* Δ*pldA* with overexpression of PBP1A or a dominant-negative elongasome subunit, PBP2(S326A) (25 μM of IPTG). Overexpression of both proteins resulted in a coccoid phenotype, indicating they inhibit the elongasome in this strain background. Microscopy was imaged at 100× magnification, and all fields of view were resized identically with a 10-μm scale bar on the first image of each panel. A single cell from the field of view is highlighted with a 2× magnified inset. (B) Doubling time of 19606 Δ*mlaE* Δ*pldA* with overexpression of PBP1A or the dominant-negative elongasome subunit PBP2(S326A). Both had no significant difference from the empty plasmid control (pMMB). Above is shown the average doubling times and significance, assessed from triplicate cultures as described in the Materials and Methods; NS indicates not significant. (C) Linear relationship between CFU/mL and OD_600_ of logarithmically growing strains with various cell morphologies of strains in this study. Cell shape changes explored in this study do not alter OD_600_ readings. Download FIG S7, PDF file, 0.2 MB. Copyright © 2021 Simpson et al. 2021 Simpson et al. https://creativecommons.org/licenses/by/4.0/ This content is distributed under the terms of the Creative Commons Attribution 4.0 International license.

## DISCUSSION

Peptidoglycan synthesis is a major target of antibiotic treatment, and it is critical that we understand these essential processes for multidrug-resistant pathogens like A. baumannii. In many model Gram-negative bacteria, PBP1A is dispersed along the periphery of the cell and thought to synthesize peptidoglycan in patches where needed (15), whereas PBP1B localizes and synthesizes peptidoglycan at the poles (15). The loss of PBP1A or PBP1B can be tolerated in E. coli, but deletion of both is synthetically lethal, indicating that despite different localizations, they are functionally redundant (15). In A. baumannii, PBP1A may be dispersed to some extent along the cell periphery but was found to also colocalize to the septa at new forming poles (27). Decreasing PBP1A levels increased the number of septa, suggesting an unprecedented role in cell division. Here, we found that PBP1A also impacts the function of the elongasome. PBP1A was demonstrated to interact with elongasome subunits in E. coli (64) and A. baumannii (32). In A. baumannii, we have observed that high levels of PBP1A caused cell rounding suggesting that it inhibited the elongasome. In agreement, Kang et al. observed that 17978 Δ*ponA* mutants were hyperresistant to mecillinam, suggesting that elongasome activity was increased when PBP1A was absent (27). Together, these findings support the idea that PBP1A could have a role in regulating or impacting two peptidoglycan synthesis processes, namely, elongation and division.

It is still unclear if PBP1A regulates or impacts elongation and division due to a direct interaction with other complexes to regulate their activity or if high PBP1A outcompetes other complexes for the lipid II precursor pool. If the morphological changes with PBP1A modulation were due to competition for lipid II precursors, then the 17978 Δ*ponA* mutant would have been expected to have an increase in both elongasome and divisome synthesis, as competition was reduced. However, 17978 Δ*ponA* was shown previously to be hyperresistant to elongasome inhibition by mecillinam but hypersensitive to divisome inhibition by aztreonam (27, 65). These opposite effects on sensitivity to elongasome and divisome inhibition support the idea that PBP1A levels could be either directly or indirectly regulating other synthesis complexes, although further proof would be required. It is possible that when PBP1A levels are low, the enzyme localizes predominantly to septa to assist with cell division, but when PBP1A levels increase, more protein may be localized to the length of the cell where it can interact with the elongasome. An increased interaction of PBP1A with the elongasome could signal for a need to reduce elongation to balance it with cell division. Strikingly, cell measurements of 17978 Δ*ponA* and 19606 wild type had no significant differences, suggesting that there was a low level of PBP1A protein that could be produced that was not inhibitory to the elongasome. This would suggest that a threshold of PBP1A protein levels has to be reached to inhibit or outcompete the elongasome.

Whether or not PBP1A levels are dynamic in A. baumannii strains and regulated for certain environments remains to be seen. Most *Gammaproteobacteria* regulate the activity of their class A PBPs with outer membrane lipoproteins. E. coli produces the activators LpoA and LpoB that activate PBP1A and PBP1B, respectively (66, 67). Pseudomonas aeruginosa, which is more closely related to A. baumannii, has an LpoA paralog that activates PBP1A but uses a unique lipoprotein activator, LpoP, to activate PBP1B (68). Notably, A. baumannii carries an *lpoP* paralog but does not have any obvious *lpoA*. It is possible that A. baumannii instead regulates PBP1A production or turnover and does not require an activator. In agreement, we have observed that overexpression of A. baumannii PBP1A alone is sufficient to cause changes in morphology. We hope to further explore how, why, and whether A. baumannii regulates PBP1A, as several open questions remain.

Studying LOS-deficient A. baumannii also unraveled novel peptidoglycan recycling genes. ElsL is an l,d-transpeptidase domain-containing protein with a previously unknown function. Disruption of *elsL* caused cell elongation defects and sensitivity to sulbactam, which are two phenotypes that mimicked elongasome mutants (32). ElsL was also found to become essential when PBP1A was deleted and during LOS deficiency (27). It was proposed that ElsL may cross-link OM proteins to the peptidoglycan layer, similar to LdtA, LdtB, and LdtC in E. coli. However, this hypothesis completely ignored that ElsL was cytoplasmic and its effect on elongasome function. Muropeptide analysis showed that *elsL* mutants had a similar increase in l,d-cross-links to wild-type cells treated with the elongasome inhibitor mecillinam. We hypothesized that ElsL was a new class of l,d-carboxypeptidases, which would explain its cytoplasmic localization and why A. baumannii does not contain other homologs of l,d-carboxypeptidases. l,d-carboxypeptidases are involved in recycling peptidoglycan fragments and have the critical role of cleaving tetrapeptides into tripeptides (15). In the absence of l,d-carboxypeptidases, tetrapeptide recycling becomes aberrant and causes a bypass of MurF addition of the fourth and fifth d-ala (57). The resulting peptidoglycan precursors with tetrapeptides cannot be utilized by d,d-transpeptidases like PBP2 of the elongasome. In A. baumannii, inactivation of the l,d-carboxypeptidase ElsL appears to inhibit peptidoglycan synthesis by the elongasome, as cells lose their rod shape. Further experimentation would be necessary to conclude if the divisome was similarly affected. In E. coli, where the elongasome is essential, inactivation of the l,d-carboxypeptidase LdcA causes stationary-phase lysis (54). Our genetic evidence supports that ElsL is a new class of l,d-carboxypeptidase. Although, biochemical assays and/or reconstitution would be necessary to prove that ElsL has this enzymatic function. Taxonomy suggests that the ElsL family of l,d-carboxypeptidase may be more widely spread but is found in several orders of *Gammaproteobacteria*. In addition, many of the organisms with an ElsL homolog are missing homologs of other l,d-carboxypeptidases, namely, LdcA and LdcV.

LPS/LOS in the OM are essential in most Gram-negative bacteria, but the reason why has remained elusive. Organisms like A. baumannii that can survive with a complete absence of LPS/LOS serve as useful tools to study the cellular role of LPS/LOS. The OM was first proposed to be a mechanical barrier because lambda phages have to disrupt both peptidoglycan and the OM in order to lyse their E. coli host when grown with high levels of divalent cations that stabilize the OM (6, 7). Biophysical experiments, predominantly in E. coli, have provided strong evidence that LPS provides rigidity to the OM to resist external forces like bending, stretching, and indentation (4). These finding were found to broadly apply to Pseudomonas aeruginosa and Vibrio cholerae as well (4) and challenged the traditional paradigm that the peptidoglycan layer was the principle mechanical component of the cell envelope. Structural capacities of peptidoglycan are evident and measurable (13, 69). Our Tn-seq and genetic analysis of LOS-deficient A. baumannii has also supported the conclusion that both the OM and peptidoglycan provide compensating structural capacities to the cell envelope.

In the absence of LOS in the OM, A. baumannii cells had severe growth defects and abnormal morphology (26, 36). We previously found that the growth and cell morphology defects of LOS-deficient A. baumannii were rescued by allowing GPLs to fill the OM (36). Inactivating the Mla and PldA pathways that normally remove or degrade GPLs from the OM outer leaflet allowed cells to grow more robustly. These data already suggested that the integrity of OM is critical for stabilizing the cell envelope. Here, we started with an Δ*mla* Δ*pldA* LOS-deficient strain that was able to survive with an OM composed of solely GPLs. Tn-seq in this strain found that key aspects of peptidoglycan synthesis and remodeling pathways had become vital for fitness. While the elongasome was not essential in LOS-containing A. baumannii, it was essential for cell integrity in LOS-deficient strains. *mla* and *pldA* suppressors in LOS-deficient mutants allowed better growth with elongasome inhibition but still cannot maintain the integrity of the cell. This finding suggested that even a stable GPL OM was not sufficient to provide the stability of an LOS-containing OM. Our work supports a recent paradigm shift indicating that cell envelope rigidity is not solely contributed by peptidoglycan but is provided by the coordination of both the asymmetrical OM and peptidoglycan. Thus, the evolution of LPS and OM asymmetry not only provides a far superior permeability barrier but also increases the structural integrity of the cell.

## MATERIALS AND METHODS

### Strains and growth conditions.

All strains and plasmids used in this study are listed in Data Set S1 in the supplemental material. Details on strain construction and growth conditions are provided in the supplemental material.

10.1128/mBio.03099-21.8 DATA SET S1 Strains, plasmids, and primers used in the manuscript. (Tab 1) Strains and plasmids used in this study. Strains that were assessed by next-generation sequencing (NGS) and the method used are indicated. (Tab 2) Primers used in this study. Download Data Set S1, XLSX file, 0.02 MB. Copyright © 2021 Simpson et al. 2021 Simpson et al. https://creativecommons.org/licenses/by/4.0/ This content is distributed under the terms of the Creative Commons Attribution 4.0 International license.

### Selecting for and confirming LOS-deficient mutants.

To facilitate high-throughput selections for LOS-deficient mutants, a 96-well microtiter plate method of selection was developed as described in the supplemental material. LOS-deficient mutants were confirmed by resistance to 10 μg/mL of polymyxin B and sensitivity to 10 μg/mL of vancomycin and assessment of LOS or lipid A levels as described in the supplemental material.

### Microscopy and cell measurements.

Overnight cultures were diluted into fresh 5-mL cultures of appropriate media at an optical density at 600 nm (OD_600_) of ∼0.05. Strains were grown to an OD_600_ of ∼0.5 to 0.7 and 8 μL was spotted onto 1-mm-thick 2% to 3% agarose pads. Cells were imaged at ×100 magnification on an Olympus CX43 phase-contrast microscope with an Infinity 5 camera and Infinity Analyze software (Teledyne Lumenera). Calibration was performed with a 1-mm ruler and 0.01-mm divisions of a Azzota Corp. micrometer slide. Measurements were performed on >3 fields of view and >400 cells per strain with MicrobeJ (59) and analyzed in GraphPad Prism 9. A one-way analysis of variance (ANOVA) test was performed with Brown-Forsythe and Welch tests assuming that standard deviations were not equal. Significant differences were assessed using a Games-Howell test.

### Assessing growth rate and phenotypes.

Overnight cultures were diluted into 5 mL of appropriate fresh media at a starting OD_600_ of ∼0.05. For LOS-deficient mutants, the medium was supplemented with 5- to 10-μg/mL polymyxin B. Cultures were vortexed and 200 μL was transferred to wells in a 96-well flat-bottom polystyrene Costar microtiter plate (Corning) to measure OD_600_ on a Synergy H1 hybrid reader with Gen5 software (BioTek). Cultures were incubated at 37°C for 8 hours or longer. Growth curves were graphed with logarithmic Y-scales, and doubling times were calculated at the fastest slope. Significant differences were tested in GraphPad Prism 9 using unpaired two-tailed *t* tests with Welch’s correction assuming standard deviations were not equal.

For mecillinam inhibition experiments, overnight cultures were diluted into 6 mL of LB at a starting OD_600_ of ∼0.05. Where appropriate, sucrose was supplemented at 146 mM (5% [wt/vol]). Cultures were incubated at 37°C, and the OD_600_ was measured each hour as described above. After the 2-hour growth time point, mecillinam was added at a final concentration of 1,000 μg/mL. Growth effects were monitored for 16 hours of total growth time. To determine if LOS-deficient mutants had reverted their LOS-inactivating mutations during the experiment, 4 μL of culture was spotted onto LB agar with 10-μg/mL vancomycin and incubated at 37°C. Growth on vancomycin indicated reversion, and these replicates were discarded and repeated.

### Microtiter live-dead assays.

Mecillinam inhibition experiments were set up as described above. At 4, 8, 12, and 16 hours of growth, 100 μL of cells was set aside and diluted in LB to an OD_600_ of 0.2 (∼2 × 10^8^ CFU/mL). A total of 100 μL of the diluted sample was labeled with 100 μL of a 2× mixture of SYTO9 and propidium iodide from the Live/Dead BacLight bacterial viability kit (catalog number L7012; Invitrogen) following manufacturer guidelines. SYTO9 and propidium iodide fluorescence were measured on a Synergy H1 hybrid reader with Gen5 software (BioTek). The ratio of live:dead signal was used to calculate the percentage of cell death using a standard curve as described in the supplemental material.

### Muropeptide analysis.

Triplicate overnight cultures were diluted in fresh 50 mL of LB at a starting OD_600_ of ∼0.05. For copper (II)- and mecillinam-treated samples, cultures were supplemented with 3.125 mM CuCl_2_ or 250 μg/mL of mecillinam. Strains were grown to an OD_600_ of ∼0.50 to 0.70 and pelleted at 5,000 × *g* for 7 min. Cells were washed with 25 mL, followed by 5 mL of 1× phosphate-buffered saline (PBS), and then pelleted after each wash. Washed pellets were resuspended in 3 mL of 1× PBS. Cells were added dropwise while stirring to boiling 3 mL of 1× PBS with 5% (wt/vol) SDS prepared fresh. Suspensions were boiled for 1 hour with stirring. Boiled lysates were switched to a room temperature stir plate and continued to stir overnight. SDS was removed by several ultracentrifugation steps, and the insoluble fraction left was resuspended in Milli-Q water. The sacculi obtained from all the cultures were treated with pronase E and muramidase, hence solubilizing the muropeptides. All reactions were inactivated by incubation at 100°C for 5 minutes. Soluble muropeptides were reduced and their pH adjusted as described previously (70) and subsequently separated by ultraperformance liquid chromatography (UPLC). The identities of the different muropeptides were analyzed using matrix-assisted laser desorption ionization–time of flight mass spectrometry (MALDI-TOF MS). GraphPad Prism 8 software was used for graphing data and statistical analysis.

10.1128/mBio.03099-21.8 TEXT S1 Supplemental materials and methods. Download Text S1, PDF file, 0.1 MB. Copyright © 2021 Simpson et al. 2021 Simpson et al. https://creativecommons.org/licenses/by/4.0/ This content is distributed under the terms of the Creative Commons Attribution 4.0 International license.

### Data availability.

Reference genomes were deposited to NCBI with accession numbers indicated in Data Set S1. All data related to this paper are within the text or supplemental material or may be requested from the authors.
