# Supplementary material for: Acinetobacter baumannii Can Survive with an Outer Membrane Lacking Lipooligosaccharide Due to Structural Support from Elongasome Peptidoglycan Synthesis
Source: mBio. 2021 Nov 30;12(6):e03099-21. doi: 10.1128/mBio.03099-21 (PMC8630537; doi:10.1128/mBio.03099-21)
Supplement: FIG S1 [file mbio.03099-21-sf001.pdf]

A

Loss of fitness during LOS-deficiency (disrupted in LOS+, not in LOS–)	
# genes	function
15	peptidoglycan synthesis/remodeling
7	cell division
8	regulation
12	OMPs, periplasmic, lipoproteins
10	inner membrane proteins
19	cytoplasmic

Gain of fitness during LOS-deficiency (disruptions enriched in LOS–)	
# genes	function
16	LOS biogenesis
2	regulation
5	cell envelope
11	cytoplasmic

B

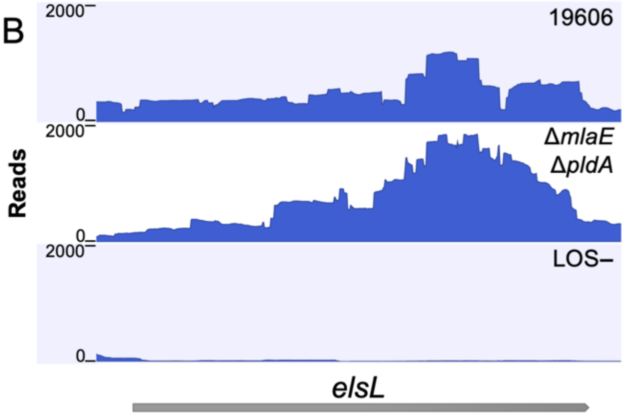

C

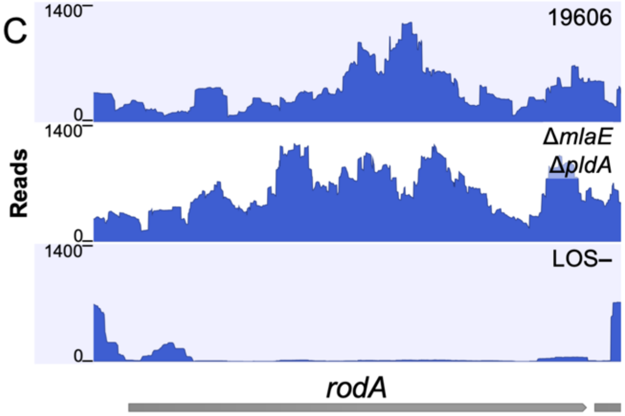

D

		19606 *vs.* LOS–		Δ*mlaE* Δ*pldA* *vs.* LOS–	
**Gene**	**Predicted function**	**FC**	***p* value**	**FC**	***p* value**
**No change in fitness LOS+ *vs.* LOS– Other non-essential peptidoglycan synthesis genes**					
ponA	PBP1A, class A penicillin binding protein	1.25	0.01	1.54	4.01E-14
ponB	PBP1B, class A penicillin binding protein	-1.39	1.47E-05	-2.13	7.11E-30
mtgA	mono-functional, glycosyltransferase	-1.39	0.23	-1.08	0.68

E

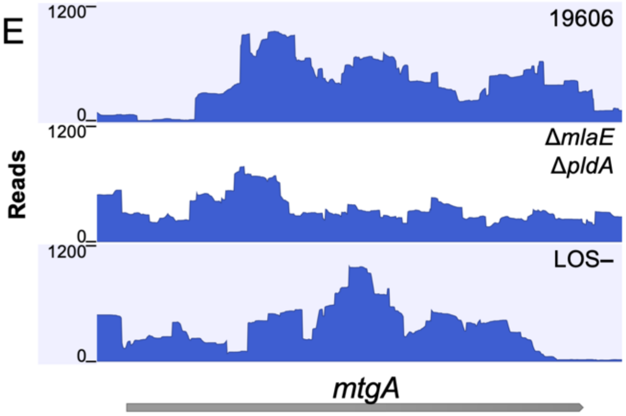

Fig. S1. Tn-seq during LOS-deficiency. A) Saturated Tn libraries were generated in an LOS-deficient strain and LOS-containing parent strains. Deep sequencing identified genes that when disrupted had a significant (≥5-fold) loss of fitness (left) or gain of fitness (right) during LOS-deficiency. Summary of gene categories for all hits. B-C) Graphical representation of read depth across representative genes with scale on left. Representative genes, *elsL* (B) and *rodA* (C), whose disruptions result in a significant loss of fitness in an LOS-deficient strain. Representative gene, *mtgA* (E), with no significant difference in Tn insertions in an LOS-deficient strain. D) non-essential peptidoglycan synthesis genes that had no change in fitness (<5 fold) between LOS-containing and LOS-deficient strains. FC indicates fold change.
