# Supplementary material for: Acinetobacter baumannii Can Survive with an Outer Membrane Lacking Lipooligosaccharide Due to Structural Support from Elongasome Peptidoglycan Synthesis
Source: mBio. 2021 Nov 30;12(6):e03099-21. doi: 10.1128/mBio.03099-21 (PMC8630537; doi:10.1128/mBio.03099-21)
Supplement: FIG S2 [file mbio.03099-21-sf002.pdf]

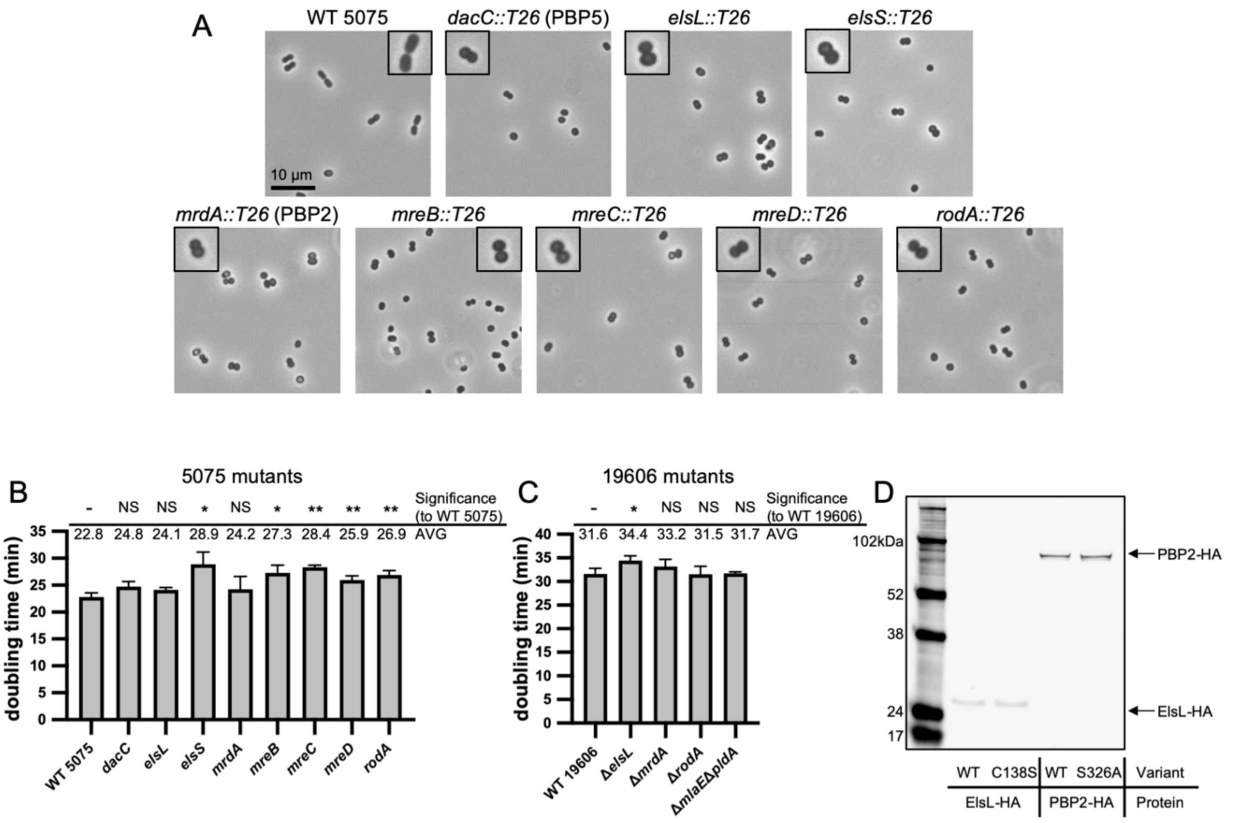

E

Inhibitor	Target(s)	WT 19606	19606 Δ*mlaE* Δ*pldA*	OM permeability fold change (WT v Δ*mlaE* Δ*pldA*)	19606 Δ*mlaE* Δ*pldA* LOS–	LOS– fold change (parent v Δ*mlaE* Δ*pldA* LOS–)	19606 LOS–
Mecillinam	PBP2	4 ± 0 mg/mL	>4 mg/mL	No effect	0.5 ± 0 mg/mL	>8x	0.25 ± 0 mg/mL
A22	MreB	125 ± 0 µg/mL	125 ± 0 µg/mL	No effect	31.3 ± 0 µg/mL	4x	31.3 ± 0 µg/mL
$CuCl_2$	ElsL, Ldt's, lipoprotein biogenesis, disulfide bonds	6.25 ± 0 mM	6.25 ± 0 mM	No effect	3.13 ± 0 mM	2x	3.13 ± 0 mM
Rifampicin	RNA pol. Blocked by LOS	1000 ± 0 ng/mL	31.3 ± 0 ng/mL	32x	0.977 ± 0 ng/mL	32x	0.977 ± 0 ng/mL

Fig. S2. All subunits of the elongasome are required for rod-shape. A) Phase contrast microscopy of 5075 Tn mutants that disrupt the elongasome. Microscopy imaged at 100x magnification and all field of views were resized identically with a 10 μm scale bar on the first image of each panel. A single cell from the field of view is highlighted with a 2X magnified inset. B) Doubling times of 5075 Tn mutants that disrupt the elongasome. C) Doubling time of deletion mutants built in 19606. Above each, average doubling times and significant differences assessed compared to respective wild type from triplicate cultures as described in methods. * indicates $p \leq 0.05$, ** indicates $p \leq 0.01$, *** indicates $p \leq 0.001$, and **** indicates $p \leq 0.0001$. D) Western blot of HA-tagged ElsL and PBP2 variants expressed from the pMMB67EHtet plasmid. E) Minimum inhibitory concentration of inhibitors that target peptidoglycan synthesis enzymes with standard deviations indicated from triplicate experiments. Rifampicin included as a drug whose influx is blocked when LOS is present in the OM. Fold-change of antibiotic sensitivity comparing 19606 and 19606 Δ*mlaE* Δ*pldA* demonstrates the effect on drug permeability due to loss of OM asymmetry. Fold-change of antibiotic sensitivity comparing the LOS-containing parent strain, 19606 Δ*mlaE* Δ*pldA*, to its LOS-deficient mutant, 19606 Δ*mlaE* Δ*pldA* LOS–, demonstrates hypersensitivities due to the loss of all LOS in the OM.
