# Supplementary material for: Acinetobacter baumannii Can Survive with an Outer Membrane Lacking Lipooligosaccharide Due to Structural Support from Elongasome Peptidoglycan Synthesis
Source: mBio. 2021 Nov 30;12(6):e03099-21. doi: 10.1128/mBio.03099-21 (PMC8630537; doi:10.1128/mBio.03099-21)
Supplement: FIG S3 [file mbio.03099-21-sf003.pdf]

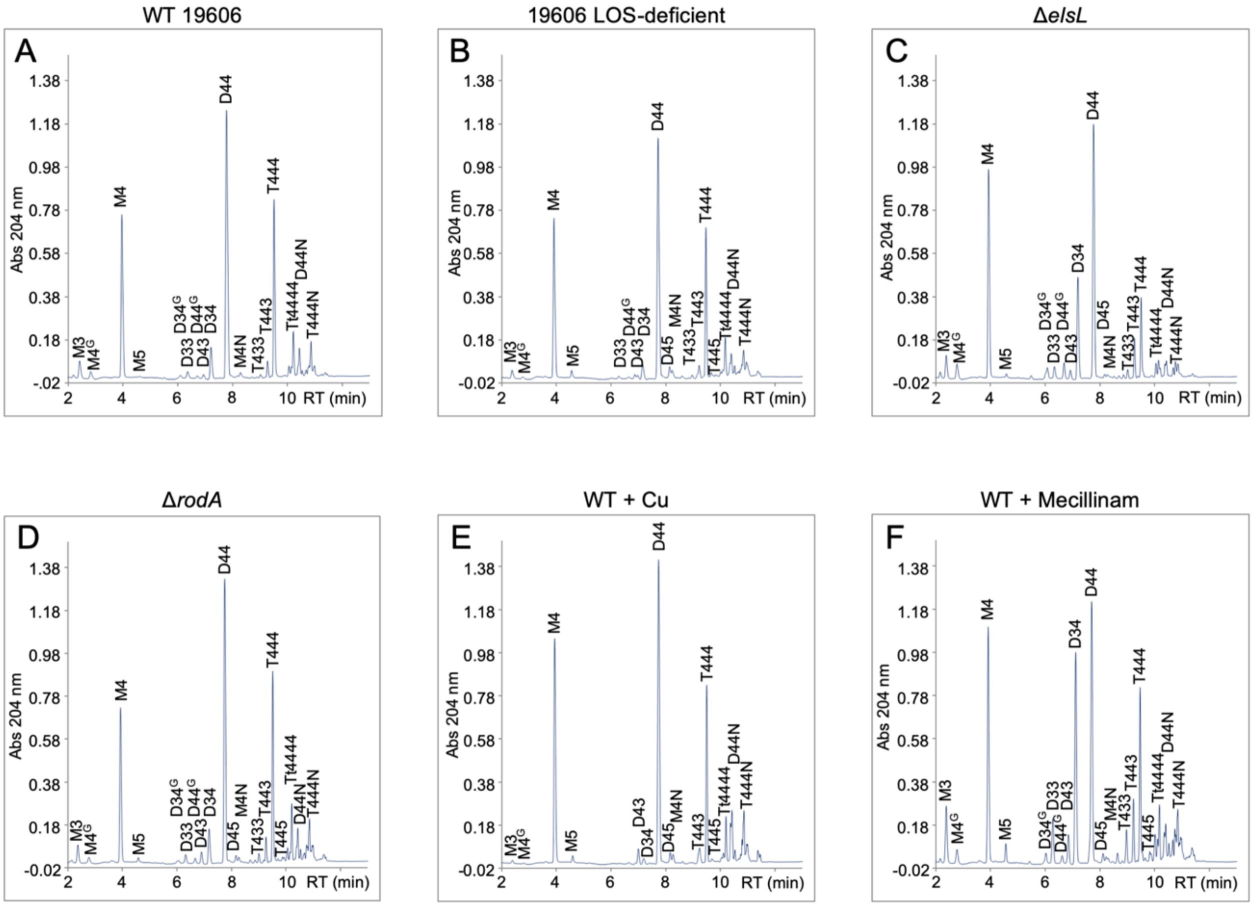

Fig. S3. Muropeptide analysis of *A. baumannii* 19606 strains. UPLC chromatograms of muropeptides from 19606 wild type (A), 19606 LOS-deficient (B), 19606 Δ*elsL* (C), and 19606 Δ*rodA* (D) grown to mid log in LB. UPLC of muropeptides from wild-type treated with 3.12 mM of copper(II) (E, abbreviated as Cu) or treated with 250 μg/mL of mecillinam (F). Muropeptides are labelled: M3, monomer disaccharide tripeptide; $M4^G$, monomer disaccharide tetrapeptide with *Gly* at fourth position; M4, monomer disaccharide tetrapeptide; M5, monomer disaccharide pentapeptide; $D34^G$, dimer disaccharide tri-tetrapeptide with one Gly at fourth position; D33, dimer disaccharide tri-tripeptide; $D44^G$, dimer disaccharide tetra-tetrapeptide with one Gly at fourth position; D43, dimer disaccharide tetra-tripeptide; D34, dimer disaccharide tri-tetrapeptide; D44, dimer disaccharide tetra-tetrapeptide; D45, dimer disaccharide tetra-pentapeptide; M4N, anhydrous monomer disaccharide tetrapeptide; T433, trimer disaccharide tetra-tri-tripeptide; T443, trimer disaccharide tetra-tetra-tripeptide; T444, trimer disaccharide tetra-tetra-tetrapeptide; T445, trimer disaccharide tetra-tetra-pentapeptide; Tt4444, tetramer disaccharide tetra-tetra-tetra-tetrapeptide; D44N, anhydrous dimer disaccharide tetra-tetrapeptide; T444N, anhydrous trimer disaccharide tetra-tetra-tetrapeptide.
