# Supplementary material for: Acinetobacter baumannii Can Survive with an Outer Membrane Lacking Lipooligosaccharide Due to Structural Support from Elongasome Peptidoglycan Synthesis
Source: mBio. 2021 Nov 30;12(6):e03099-21. doi: 10.1128/mBio.03099-21 (PMC8630537; doi:10.1128/mBio.03099-21)
Supplement: FIG S4 [file mbio.03099-21-sf004.pdf]

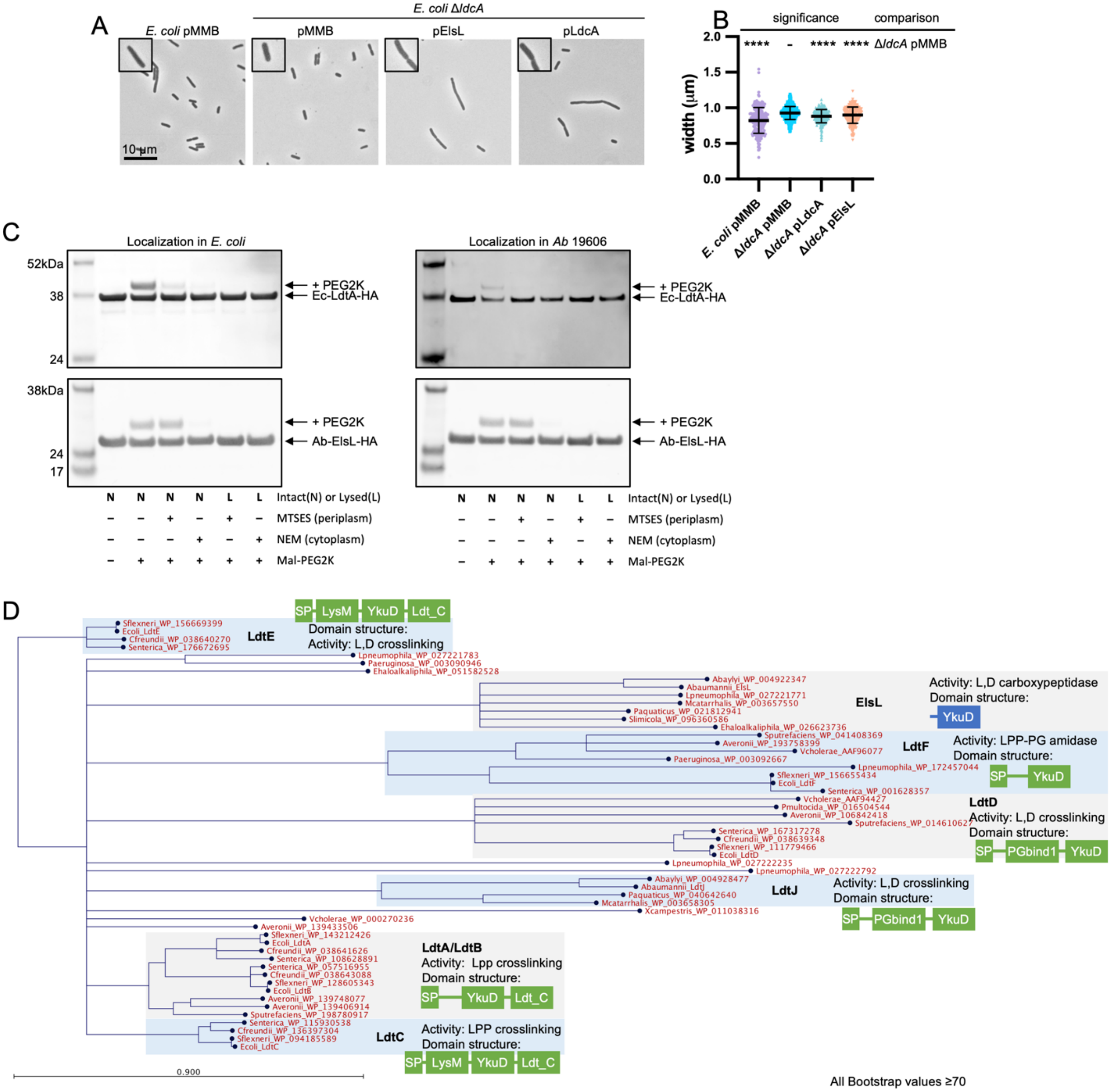

Fig. S4. ElsL belongs to a new family of L,D-carboxypeptidases. A-B) *A. baumannii* ElsL complements cellular morphology changes of an *E. coli* Δ*ldcA* mutant. Phase contrast microscopy (A) and cell measurements (B) of *E. coli* Δ*ldcA* mutants with empty (pMMB) or complementation plasmids encoding ElsL from *A. baumannii* or LdcA, from *E. coli*. Microscopy imaged at 100x magnification and all field of views were resized identically with a 10 μm scale bar on the first image of each panel. A single cell from the field of view is highlighted with a 2X magnified inset. Cell measurements performed on ≥400 cells with MicrobeJ and assessed for significant differences as indicated in methods, * indicates $p \leq 0.05$, ** indicates $p \leq 0.01$, *** indicates $p \leq 0.001$, and **** indicates $p \leq 0.0001$. C) Substituted-cysteine accessibility method to assess localization of *E. coli* LdtA (top) and *A. baumannii* ElsL (bottom) in whole cells. Intact (N) cells were treated

with cysteine-reactive compounds that either can (NEM) or cannot (MTSES) permeate through the inner membrane. Presence or absence of modifications were assessed after lysing cells and denaturing by the ability to block cysteine modification by a 2kDa mal-PEG (Mal-PEG2k). D) Maximum likelihood phylogeny of L,D-transpeptidase domain-containing proteins found in gamma-proteobacteria. Tree was tested with 100 bootstrap iterations. Branches with known function homologs are highlighted and the homolog's name, function, and domain architecture are shown. SP indicates the presence of a signal peptide predicted by SignalP5.0 (11). All other domains refer to Pfam (12) domains: YkuD=PF03734, L,D-transpeptidase catalytic domain; LysM=PF01476, LysM domain; Ldt_C=PF17969, L,D-transpeptidase C-terminal domain; PGbind1=PF01471, Putative peptidoglycan binding domain.
