# Supplementary material for: Acinetobacter baumannii Can Survive with an Outer Membrane Lacking Lipooligosaccharide Due to Structural Support from Elongasome Peptidoglycan Synthesis
Source: mBio. 2021 Nov 30;12(6):e03099-21. doi: 10.1128/mBio.03099-21 (PMC8630537; doi:10.1128/mBio.03099-21)
Supplement: FIG S5 [file mbio.03099-21-sf005.pdf]

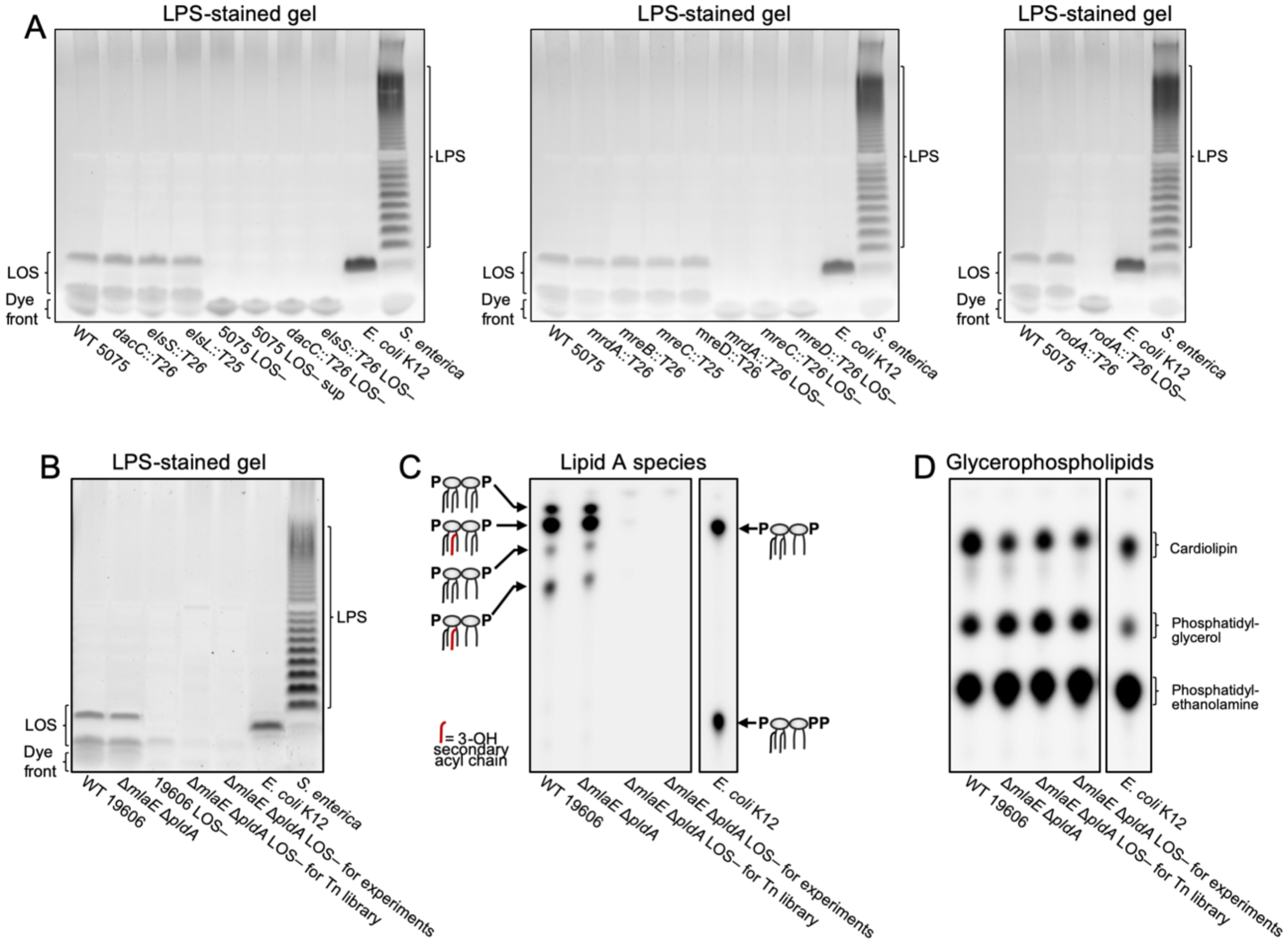

Fig. S5. LOS-deficient strains produce no LOS. A) Gel electrophoresis and ProQ emerald stain of LOS isolated from 5075 Tn mutants that disrupt the elongasome and LOS-deficient mutants isolated from these strains. *E. coli* W3110 (labeled *E. coli* K12) and *S. enterica* serovar typhimurium LT2 (labeled *S. enterica*) used as controls for LOS and LPS staining respectively. Since *E. coli* K12 strains lack O-antigen, the LPS of these bacteria has similar size to LOS. B) Gel electrophoresis and ProQ emerald stain of LOS isolated from 19606 LOS-deficient strains: 19606 LOS–, the Δ*mlaE* Δ*pldA* LOS-deficient strain used for building a Tn-mutant library (labeled Δ*mlaE* Δ*pldA* LOS– for Tn library) and the Δ*mlaE* Δ*pldA* Δ*lpxC* LOS-deficient strain used for experiments (labeled Δ*mlaE* Δ*pldA* LOS– for experiments). C-D) TLC analysis of lipid A species (C) and glycerophospholipids (D) from19606 LOS-deficient strains labeled with $^{32}P_i$.
