# Supplementary material for: Acinetobacter baumannii Can Survive with an Outer Membrane Lacking Lipooligosaccharide Due to Structural Support from Elongasome Peptidoglycan Synthesis
Source: mBio. 2021 Nov 30;12(6):e03099-21. doi: 10.1128/mBio.03099-21 (PMC8630537; doi:10.1128/mBio.03099-21)
Supplement: FIG S6 [file mbio.03099-21-sf006.pdf]

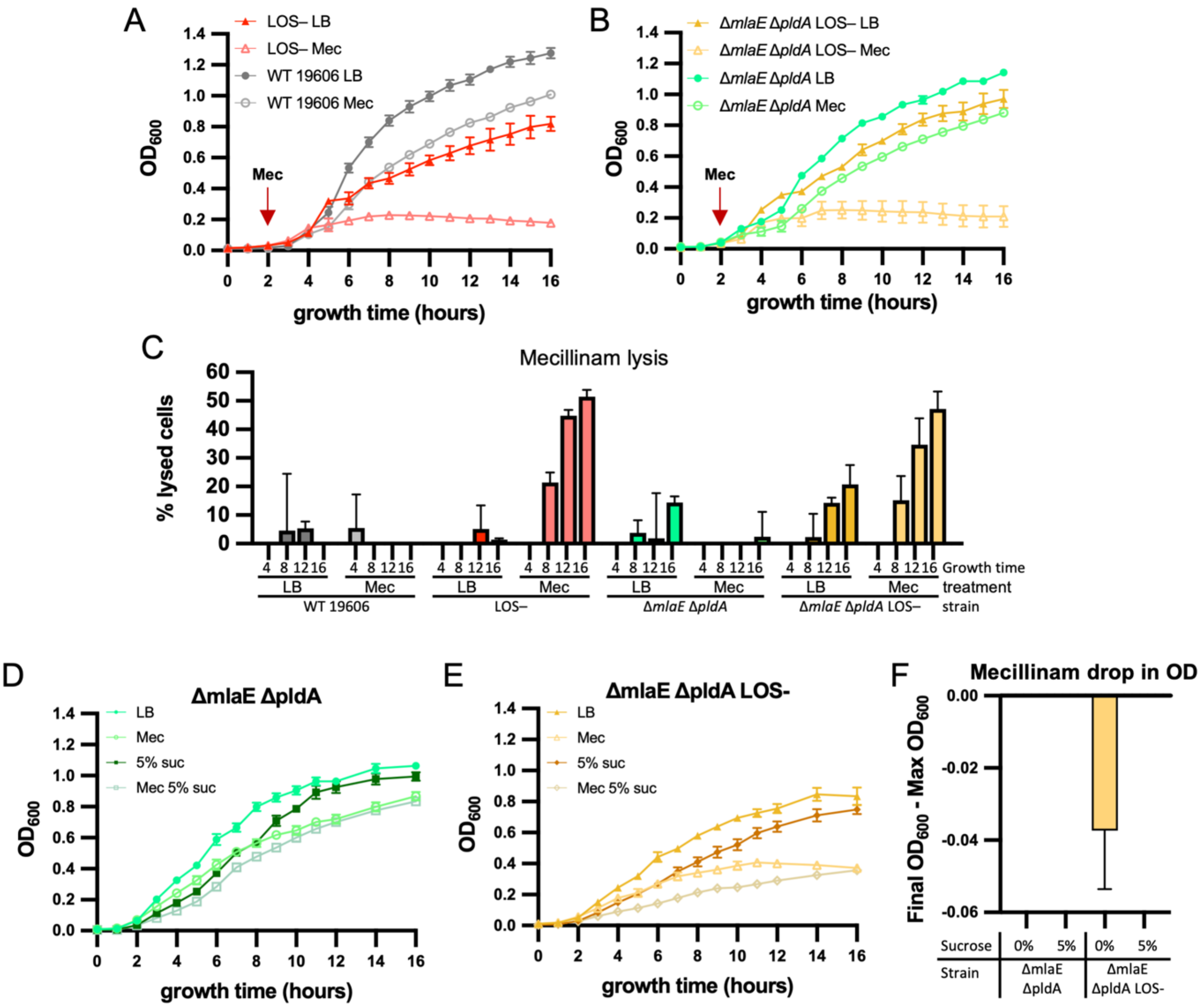

Fig. S6. Disruption of the elongasome during LOS-deficiency results in cell lysis. Chemical inhibition of PBP2 of the elongasome with 1000 μg/mL of mecillinam of actively growing (added after 2 hours of growth) LOS-containing or LOS-deficient *A. baumannii* 19606 strains. A) Averages of triplicate growth curves of mecillinam-inhibited (open symbols) compared to LB cultured (closed symbols) for the wild-type 19606 (circles) and 19606 LOS-deficient strain (triangles). B) Averages of triplicate growth curves of mecillinam-inhibited (open symbols) compared to LB cultured (closed symbols) for the 19606 Δ*mlaE* Δ*pldA* (circles) and 19606 Δ*mlaE* Δ*pldA* LOS-deficient strain (triangles). C) Microtiter live-dead assays to assess the amount of cell lysis with prolonged exposure to mecillinam. % lysed cells calculated from a standard curve of intact and lysed cells of the wild-type 19606 strain as described in methods. D-F) Increasing external osmotic pressure, 5% sucrose (suc), protects mecillinam-inhibited LOS-deficient cells from lysing. Mecillinam-inhibited (open symbols) cultures compared to LB cultures (closed symbols) with an without 5% sucrose of 19606 Δ*mlaE* Δ*pldA* (D) and 19606 Δ*mlaE* Δ*pldA* LOS-deficient (E) strains. F) Calculated decline in OD_{600} during mecillinam treatment.
