# Supplementary material for: Acinetobacter baumannii Can Survive with an Outer Membrane Lacking Lipooligosaccharide Due to Structural Support from Elongasome Peptidoglycan Synthesis
Source: mBio. 2021 Nov 30;12(6):e03099-21. doi: 10.1128/mBio.03099-21 (PMC8630537; doi:10.1128/mBio.03099-21)
Supplement: FIG S7 [file mbio.03099-21-sf007.pdf]

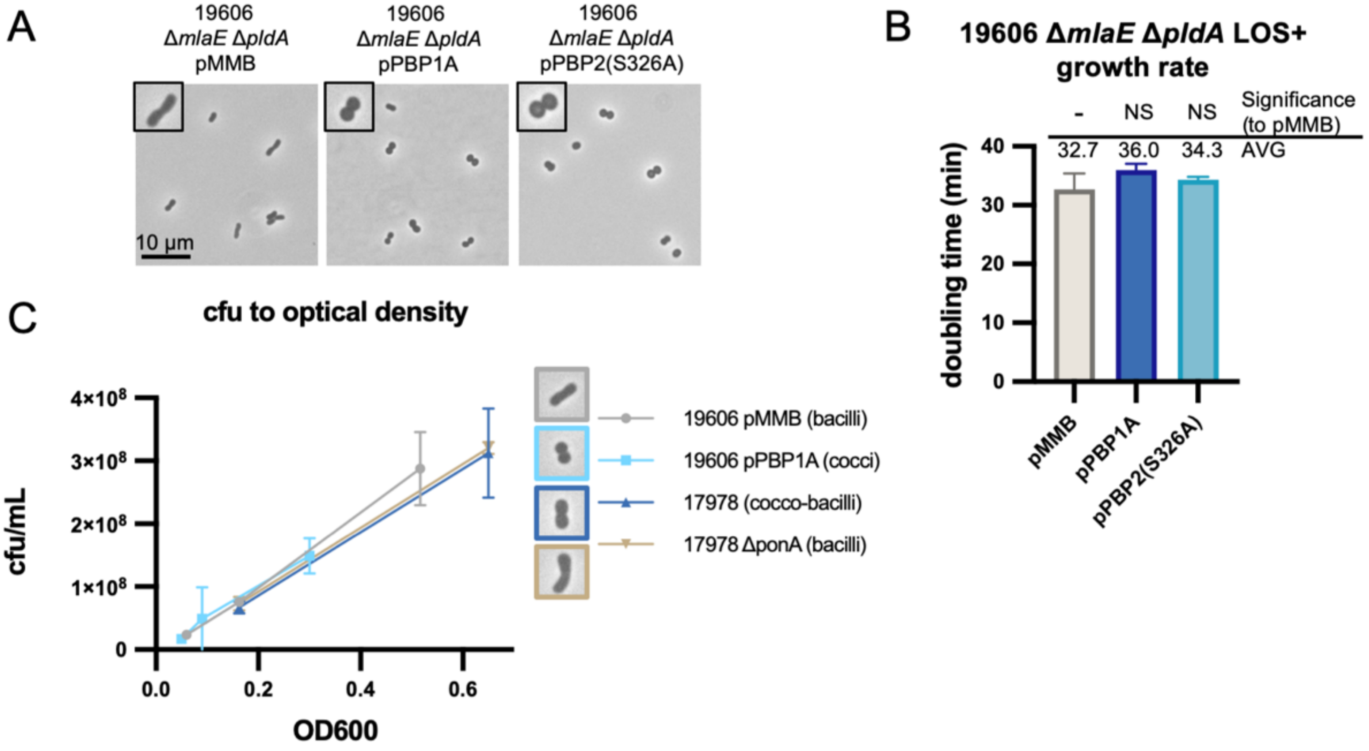

Fig. S7. Overexpression of PBP1A inhibits the elongasome, but does not affect growth rate of LOS-containing *A. baumannii*. A) Phase contrast microscopy of 19606 Δ*mlaE* Δ*pldA* with overexpression of PBP1A or a dominant-negative elongasome subunit, PBP2(S326A) (25 μM of IPTG). Overexpression of both proteins resulted in a coccoid phenotype, indicating they inhibit the elongasome in this strain background. Microscopy imaged at 100x magnification and all field of views were resized identically with a 10 μm scale bar on the first image of each panel. A single cell from the field of view is highlighted with a 2X magnified inset. B) Doubling time of 19606 Δ*mlaE* Δ*pldA* with overexpression of PBP1A or the dominant-negative elongasome subunit, PBP2(S326A). Both had no significant difference from the empty plasmid control (pMMB). Above, average doubling times and significance, assessed from triplicate cultures as described in the methods, NS indicates not significant. C) Linear relationship between cfu/mL and OD_{600} of logarithmically growing strains with various cell morphologies of strains in this study. Cell shape changes explored in this study do not alter OD_{600} readings.
