# Supplementary material for: Acinetobacter baumannii Can Survive with an Outer Membrane Lacking Lipooligosaccharide Due to Structural Support from Elongasome Peptidoglycan Synthesis
Source: mBio. 2021 Nov 30;12(6):e03099-21. doi: 10.1128/mBio.03099-21 (PMC8630537; doi:10.1128/mBio.03099-21)
Supplement: TEXT S1 [file mbio.03099-21-t0001.pdf]

Supplemental Materials and Methods

Media and growth conditions. All cultures and agar plates were performed with Difco™ Luria-Bertani Broth or Agar, Miller (BD). Where appropriate media was supplemented with kanamycin (30 μg/mL), tetracycline (5-10 μg/mL), polymyxin B (10 μg/mL unless otherwise indicated), and vancomycin (10 μg/mL unless otherwise indicated). Concentration of other antibiotics are indicated in the respective experiments.

Strains and plasmids. All strains and plasmids used in this study are listed in Data Set S1. All oligonucleotides used in this study were synthesized by IDT and are listed in Data Set S1. *A. baumannii* 5075 Tn-mutants were struck from the Manoil lab Tn-library collection (1) on LB agar with 5 μg/mL of tetracycline and confirmed with primers up and downstream of the appropriate gene. *A. baumannii* 19606 deletion mutants were built by recombineering as described previously (2). We noted that all 19606 recombineered strains shared mutations found in their parent strain. The isogenic parent to 19606 recombineered strain, BWS399, was used as the 19606 "wild-type" for this study. LOS-deficient mutants were isolated as described below. *E. coli* deletion alleles were obtained from the Keio collection (3) and confirmed with primers up and downstream of the appropriate gene. Deletion alleles were transduced by $P1_{vir}$ transduction (4) into the *E. coli* W3110 wild-type strain for all characterization.

TaKaRa ExTaq® polymerase or Apex™ Taq RED polymerase were used for PCRs <5 kb for cloning or screening experiments, respectively. PFU Turbo polymerase AD (Agilent) was used for PCRs >5 kb. Complementation plasmids were built by digesting pMMB67EHtet with appropriate restriction enzymes (NEB) and treating digested plasmid with Antarctic Phosphatase (NEB). Inserts were PCR amplified with EcoRI-elsL-for and XbaI-elsL-HA-rev for the *elsL* gene from *A. baumannii* ATCC 19606; SacI-RBS-mrdA-for and XbaI-mrdA-HA-rev for the *mrdA* gene from *A. baumannii* ATCC 19606; SacI-RBS-rodA-for and XbaI-rodA-HA-rev for the *rodA* gene from *A. baumannii* ATCC 19606; EcoRI-Ec-ldcA-for and XbaI-Ec-LdcA-HA-rev for the *ldcA* gene from *E. coli* W3110; and EcoRI-ponA-for and XbaI-ponA-HA-rev for the *ponA* gene from *A. baumannii* ATCC 19606. Inserts were digested with the restriction enzymes indicated by their primer names. Insert and vectors were ligated by T4 DNA Ligase (NEB) and transformed by electroporation into DH5α competent cells. Plasmids were screened by colony PCR with the primers ptac-seq and rrnB-T1-term-seq for the correct insert size and Sanger sequenced by Genewiz® with the same primers.

The pMMB67EHtet-RodA-HA plasmid did not complement cell shape changes of a 19606 Δ*rodA* mutant, indicating that the C-terminal HA tag was likely detrimental to function. The first amino acid of the tag was changed to a stop codon by site-directed mutagenesis (5). The whole plasmid was amplified with the primers pRodA-stop-for and pRodA-stop-rev. Parent plasmid was digested with DpnI (NEB) and the PCR was electroporated into DH5α. The point mutation was confirmed by Sanger sequencing with ptac-seq and rrnB-T1-term-seq. Catalytically-inactive mutants, *elsL(C138S)* and *mrdA(S326A)*, were built by site-directed mutagenesis of pMMB67EHtet-ElsL-HA and pMMB67EHtet-MrdA-HA, respectively, using the same methodology. Primers used were elsL-C138S-for / elsL-C138S-rev and mrdA-S326A-for / mrdA-S326A-rev.

Selecting for and confirming LOS-deficient mutants. To facilitate high-throughput selections for LOS-deficient mutants, a 96-well microtiter plate method of selection was developed. 10 colonies of each strain were used to inoculate 10 replicate wells with 200 μl of LB each in a 96-well flat-bottom polystyrene costar® microtiter plate (Corning). Rows of LB only wells were used to separate each strain replicates and prevent cross-contamination. Plates were sealed with AeraSeal™ film (EXCEL Scientific, inc.) and incubated at 37 °C for 16-20 hours. 20 μl of each overnight replicate was diluted into 180 μl of LB with polymyxin B (final concentrations 10μg/mL) in a well of a 96-well round-bottom polypropylene costar®microtiter plate (Corning). Polypropylene plates were used for polymyxin B cultures because polystyrene can bind and reduce the concentration of polymyxins (6). Selection plates were sealed with AeraSeal™ film and incubated at 37 °C for 24 hours. After incubation, selection plates were well mixed by pipetting 100 μl up and down, and 4 μl was spotted on to LB agar plates with 10 μg/mL of polymyxin B to isolate the LOS-deficient mutants. This method of selection was confirmed to work as expected for 5075 and 17978 strains. However, 19606 strains, occasionally selected for partially reduced LOS mutants (~50% reduction) with this method. Increasing the selective pressure of polymyxin B to 15 μg/mL for 19606 strains, reduced the

rate of partially reduced LOS mutants. Frequency of LOS-deficient mutants represented in Figures includes only mutants with complete absence of LOS.

LOS-deficient mutants were confirmed by multiple metrics as describe previously (7, 8). In addition to resistance to 10 μg/mL of polymyxin B, sensitivity to 10 μg/mL of vancomycin was also checked by patching on to LB agar plates with these antibiotics. Normally LOS in the OM blocks entry of vancomycin. In the absence of LOS, cells become sensitive to vancomycin that can now enter. Finally, presence or absence of LOS was measured directly (Fig. S5) by proteinase K treating lysates, separating macromolecules with SDS-PAGE gel electrophoresis, and staining glycolipids with ProQ® Emerald 300 lipopolysaccharide staining kit (Molecular Probes, Inc.) as described previously (8).

We noted that all of our 19606 LOS-deficient mutants have a small glycolipid that stains with ProQ Emerald 300 lipopolysaccharide stain (Fig. S5B). We suspected this glycolipid was not residual lipid A because our strains have disrupting mutations in *lpxC* . To determine if this glycolipid was residual lipid A, we compared to our stable LOS-deficient strain with a complete deletion of the *lpxC* gene (19606 Δ*mlaE* Δ*pldA* Δ*lpxC lpxA::IS4*). The Δ*lpxC* strain still has this small glycolipid, indicating it is not residual lipid A (Fig. S5B). To further assess the amount of lipid A in our 19606 LOS- deficient strains, lipids were radiolabeled with 2.5 mCi of ^{32}P ortho-phosphoric acid (Perkin-Elmer) and isolated as described previously (8). Lipid A and GPLs were isolated, separated by thin layer chromatography, and imaged by phosphor screening. 19606 LOS-deficient mutants were confirmed to have complete absence of lipid A, with no changes to GPL levels (Fig. S5C-D).

Whole-genome sequencing. Strains that were whole genome sequenced are indicated in Data Set S1. We noted that our 19606 wild-type strain had many differences from the Genbank *A. baumannii* ATCC 19606 genome sequence (Accession number NZ_ACQB00000000). To get a more accurate reference genome, our 19606 wild-type strain was grown overnight in 5 mL of LB. 1 mL of culture was spun down at 13,000 x *g* for 1 min, supernatant was removed, and the pellet was stored at -20°C. The pellet was shipped to Genewiz® for DNA extraction, PacBio library preparation, PacBio Sequel whole genome sequencing and *de novo* genome assembly. The assembled genome was annotated in CLC Genomics Workbench with the Genome Finishing Module Plugin (Qiagen) by transferring annotations from the NZ_ACQB00000000 genome to the de novo assembly. Our 19606 wild-type genome was submitted to Genbank (Accession number CP074585-CP074586).

Whole-genome sequencing of mutants was performed by growing the strain overnight in 5 mL of LB. 1 mL of culture was pelleted at 13,000 x *g* for 1 min and genomic DNA was extracted using an Easy-DNA™ gDNA purification kit (Invitrogen). Genomic DNA was prepared for whole-genome sequencing using Nextera™ DNA Flex Library Prep kit (Illumina) with Nextera™ DNA CD 24-indexes (Illumina) per manufacturer instructions. Libraries were pooled and sequenced on an iSeq100 (Illumina). Reads were trimmed, locally realigned, and variants mapped using CLC Genomics Workbench.

Transposon mutagenesis and mutant library builds. Transposon mutagenesis was performed using EZ-Tn5™ transposome kits (Lucigen) per manufacturer instructions. Briefly, each strain was first assessed for transposon mutagenesis efficiency. Strains were grown overnight in 5 mL of appropriate media and diluted to a starting OD_{600}~0.05 in a fresh 50 mL culture of the same media. Strains were grown to an OD_{600}~0.5-0.7. Cells were transferred to 50 mL conical tubes (**fisher**brand), pelleted at 5,000 x *g* for 7 min, and washed twice with 10% glycerol. Pellets were resuspended in 1 mL of 10% glycerol and transferred to 1.5 mL microcentrifuge tubes (**fisher**brand). Cells were spun down at 13,000 x *g* for 5 min and resuspended in 200 μl of 10% glycerol. 50 μl of cells were mixed with 1 μl of transposomes and transferred to 0.1 cm electroporation cuvettes (Bio-Rad). Cells were electroporated at 1.8 kV with a MicroPulser™ electroporator (Bio-Rad) and immediately resuspended in 1 mL of LB. Electroporated cells were grown at 37 °C for 1.5 hours with shaking. Undiluted, 1:10, and 1:100 dilutions were plated on 100 mm LB agar plates with 30 μg/mL of kanamycin with a 1:10 plating dilution and incubated at 37 °C for 16-20 hours.

Efficiency of transposon mutagenesis was calculated for each strain and transposon mutagenesis was upscaled aiming to build a library of ~150,000-200,000 mutants. Upscaled transposon mutagenesis was performed in the same manner, increasing the number of cultures and electroporation reactions as appropriate. After outgrowth of the electroporated cells, all reactions were combined and spun down at 5,000 x *g* for 7 min. The pellet was resuspended in an appropriate volume to plate ~13,000 cfu in 150 μl. 150 μl of the resuspension was plated on the appropriate number of 150 mm (Genesee Scientific) LB agar

plates with 30 μg/mL of kanamycin for the desired library size. Plates were incubated for 14-16 hours at 37 °C. Three representative plates were counted to calculate the library size and plates were stored at 4 °C for 20-48 hours. Note, storing plates in the fridge allowed for condensation that made scraping libraries easier. However, LOS-deficient strains do not store well at 4 °C and this library was instead immediately scraped.

Transposon mutant libraries were scraped with 3.0 cm bladed cell scrapers (Falcon®) and mixed into 8 mL of LB with 30% glycerol. After scraping all plates, the cell mixture was slowly mixed with a 5 mL Finnpipette® F2 (Thermo Scientific) to homogenize the solution. As needed 70 μm cell strainers (Falcon®) were used to break up cell clumps that were resistant to pipetting. Once fully homogenized, 100 μl of library mixture was aliquoted to ~80 cryovial and the library was stored at -80°C.

Transposon sequencing and analysis. Growth challenges of Tn-mutant libraries and DNA library preparations were performed in triplicate as previously described (9) with a few modifications. In the first PCR step, the primer, biotin EZ Tn5, was used instead of olj510-Biotin and, in the second PCR step, the primer, P5 EZ Tn5 was used instead of olj511.

Samples were paired-end sequenced using an Illumina HiSeq 2500 by Genewiz®. Tn-seq data analysis was performed using CLC Genomics Workbench (Quiagen) and the *A. baumannii* ATCC 19606 genome sequence (Genbank accession number NZ_ACQB00000000). Significant hits were identified as those with a ≥5 fold difference between libraries and *p*-values ≤ 0.05. Significant hits were manually curated to remove hits whose difference in RPKM had an absolute value less than 50 (e.g. RPKM of 0.2 in one strain and 10 in a second is still indicative of essential in both strains) and to remove hits that had uneven insertions only at the 5' or 3' ends of genes (possible over expressions or truncations, respectively).

Immunoblots. Overnight cultures were back diluted to a starting OD_{600}~ 0.05 in fresh 5.5 mL of appropriate media. Cultures were grown to OD_{600}~ 0.50-0.70 and 5 mL was pelleted. Pellets were resuspended in 500μl of fresh 1x PBS with 1% SDS and boiled for 5 min. Lysates were cooled to room temperature then centrifuged at 17,000 x *g* for 10 min. Protein concentration was determined with a Pierce™BCA protein assay kit (Thermo Scientific). Lysates were diluted to 0.5 μg/μl of protein in NuPAGE® LDS sample buffer (Invitrogen) with 5% *β*-mercaptoethanol. Samples were boiled for 10 min and 10 μg of protein was run on a NuPAGE® 10% Bis-Tris gel (Invitrogen). Proteins were transferred to Amersham™ Protran™ Premium 0.45 μm nitrocellulose in a BioRad Trans-Blot® SD Semi-Dry Transfer cell in Towbin buffer. Blots were probed with primary and secondary antibodies, The™ HA-tag antibody mouse (Genscript) and Cy5™ goat anti-mouse IgG (Invitrogen) respectively, and imaged for Cy5 fluorescence on an Amersham™ Typhoon™ Biomolecular Imager.

Minimal inhibitory concentration determinations. Overnight cultures were diluted 1:1,000 in 20 mL of fresh LB per antibiotic tested. 2 mL of diluted culture was aliquoted to 8 tubes and an additional 2 mL was added to the first tube. For LOS-containing strains antibiotics were added to the first tube at a final concentration of 4000 μg/mL (mecillinam), 500 μg/mL (A22), 12.5 mM (copper(II) chloride), and 4 μg/mL (rifampicin). For LOS-deficient strains antibiotics were added to the first tube at a final concentration of 1000 μg/mL (mecillinam), 500 μg/mL (A22), 12.5 mM (copper(II) chloride), and 62.5 ng/mL (rifampicin). Each strain/antibiotic combination was serially diluted 1:2 by transferring 2 mL of tube 1 into tube 2, vortexing, transferring 2 mL from tube 2 into tube 3, vortexing, and so forth until diluted to tube 7. The extra 2 mL of media was removed from tube 7 and tube 8 served as an untreated control. Cultures were grown for 16 hours at 37 °C with shaking and terminal OD_{600} readings were measured as described for growth experiments. MIC determinations were calculated as the concentration at which 90% of growth was inhibited relative to an untreated control. To determine if LOS-deficient mutants had reverted their LOS-inactivating mutations during the time span of the experiment, 4 μl of every LOS-deficient tube was spotted on to LB agar with 10 μg/mL of polymyxin B and LB agar with 10 μg/mL of vancomycin and incubated at 37°C. Growth on LB agar with vancomycin indicated reversion of the LOS-deficiency mutations during the experiment. Replicates with reversion near the MIC were discarded and repeated.

Live-Dead assay standard curve. During the inhibition experiment, one 19606 wild-type overnight culture was diluted into fresh 50 mL of LB in a 125 mL flask for standard curve. Standard curve was prepared from logarithmically grown cells following manufacturer guideline with some adaptations. Two 50 mL conical

tubes each with 10 mL of logarithmically grown cells were pelleted at 5,000 x *g* for 7 min and resuspended in 1 mL of 10% glycerol. For a live-cells sample, 10% glycerol was added to one tube correcting to OD_{600}=0.2. An equal volume of 25% isopropanol was added to the second tube, to gently lyse cells for a dead-cell sample. Live and dead samples were incubated with gentle rocking for 1 hour at room temperature. Live and dead samples were pelleted at 5,000 x *g* for 7 min and resuspended gently in 1 mL of LB. Additional LB was added to correct each sample to OD_{600}=0.2. Live and dead samples were mixed gently at ratios of 0:100, 10:90, 25:75, 50:50, 75:25, 90:10, and 100:0 of dead:live cells in a total volume of 500 μl. Triplicate samples of each standard was stained with SYTO9 and propidium iodide per manufacturer guidelines and fluorescence was measured as described above. The ratio of green:red flurosecence was calculated for each sample and plotted to the percent of dead cells. A linear regression was calculated (R^2 <0.99) and used to calculate the percent lysis during the mecillinam inhibition experiment.

Controlling for cell shape impact on growth curves. To determine if changes in cell shape observed in this study affected OD_{600} measurements in a manner that could alter interpretations of growth curves, strains with a representative bacilli, cocci, and cocco-bacilli cellular morphology were grown to a few logarithmic cell densities and cfu/mL determinations were performed. Triplicate overnight cultures were diluted into fresh 5 mL of LB with appropriate antibiotics. Strains were grown for 3 hours and each hour a 1:10 dilution series was performed in 1 mL of LB. 100 μl of each dilution were plated on LB agar and incubated at 37 °C overnight. Cfu's were enumerated and the cfu/mL of each time point was calculated. All representative cellular morphologies had the same linear relationship between OD_{600} and cfu/mL, indicating that differences in cellular morphologies did not impact the interpretation of growth curves (Fig. S7C).

Substituted-cysteine accessibility method to assess ElsL localization. Overnight cultures were diluted into fresh 5 mL of LB with tetracycline and IPTG (50 μM for *E. coli* W3110 strains and 500 μM for *A. baumannii* 19606 strains). Lysis buffer consisting of 1x PBS with 1% SDS was prepared fresh each day. Cells were grown to OD_{600} ~1.0 at 37 °C. 3.5 mL of cells were pelleted, washed once with 1x PBS, and resuspended in a volume of 1x PBS to correct for cell density. For intact cell labeling, 4 tubes, tubes A-D, were prepared each with 50 μl of cells. For lysed cell controls, 2 tubes with 50 μl each of cells, tubes E and F, were immediately pelleted again and resuspended in 90 μl of lysis buffer. Lysed samples were boiled for 5 min, cooled to room temperature, and then centrifuged at 17,000 x *g* for 10 min. Supernatants were transferred to fresh tubes. When both intact and lysed cells were ready for blocking, 5 μl of 100 mM *N*-ethylmaleimide (NEM, Sigma Aldrich) was added to tubes D and F, and 5 μl of 100 mM sodium (2-sulfonatoethyl) methanethiosulfonate (MTSES, Cayman chemicals) was added to tubes C and E, a lysed and intact sample for each. No blockers were added to tube A and B. All tubes were incubated with gentle rocking for 1 hour at room temperature. After incubation, 500 μl of 1x PBS was added to intact cell samples, tubes A-D, to dilute the cysteine-reactive blockers. Tubes A-D were pelleted and washed once with 1x PBS. Tubes A-D were resuspended in 90 μl of lysis buffer and treated as described above to lyse and denature protein. When all samples were lysed, 2.5 μl of 5 mM maleimide-polyethylene glycol (Mal-PEG, molecular weight 2,000 Da, Laysan Bio) was added to all samples except tube A, as an unblocked and unlabeled control. Tubes were incubated with gentle rocking at room temperature for 1 hour with protection from light. 4x NuPAGE® LDS sample buffer (Invitrogen) and *β*-mercaptoethanol were added to each sample diluting to 1x and 1% respectively. Samples were boiled for 10 min and 20 μl of each sample was run on a NuPAGE® 10% Bis-Tris gel (Invitrogen). Gels were transferred and immunoblotted as described in supplemental.

ElsL taxonomy. Ldt-domain containing proteins were compiled from representative organisms from 10 common orders of the gamma-proteobacteria phylum, acidiferrobacterales, aeromonadales, alteromonadales, chromatiales, enterobacterales, legionellales, pasteurellales, pseudomonadales, vibrionales, and xanthomonadales. Sequences were aligned by MUSCLE (10) and a maximum likelihood phylogenetic tree was built by neighbor joining method in CLC genomics workbench with 100 bootstrapping iterations.

Supplemental References

1. Gallagher LA, Ramage E, Weiss EJ, Radey M, Hayden HS, Held KG, Huse HK, Zurawski DV, Brittnacher MJ, Manoil C. 2015. Resources for Genetic and Genomic Analysis of Emerging Pathogen Acinetobacter baumannii. J Bacteriol 197:2027–2035.

2. Tucker AT, Nowicki EM, Boll JM, Knauf GA, Burdis NC, Trent MS, Davies BW. 2014. Defining gene-phenotype relationships in Acinetobacter baumannii through one-step chromosomal gene inactivation. mBio 5:e01313-01314.

3. Baba T, Ara T, Hasegawa M, Takai Y, Okumura Y, Baba M, Datsenko KA, Tomita M, Wanner BL, Mori H. 2006. Construction of Escherichia coli K-12 in-frame, single-gene knockout mutants: the Keio collection. Mol Syst Biol 2:2006.0008.

4. Silhavy TJ, Berman ML, Enquist LW. 1984. Experiments with Gene Fusions. Cold Spring Harbor Lab Press, Cold Spring Harbor, NY.

5. Zheng L, Baumann U, Reymond J-L. 2004. An efficient one-step site-directed and site-saturation mutagenesis protocol. Nucleic Acids Res 32:e115.

6. Li J, Nation RL, Kaye KS. 2019. Polymyxin Antibiotics: From Laboratory Bench to Bedside. Springer Nature, Switzerland.

7. Boll JM, Crofts AA, Peters K, Cattoir V, Vollmer W, Davies BW, Trent MS. 2016. A penicillin-binding protein inhibits selection of colistin-resistant, lipooligosaccharide-deficient Acinetobacter baumannii. Proc Natl Acad Sci U S A 113:E6228–E6237.

8. Powers MJ, Trent MS. 2018. Phospholipid retention in the absence of asymmetry strengthens the outer membrane permeability barrier to last-resort antibiotics. Proc Natl Acad Sci U S A 115:E8518–E8527.

9. Douglass MV, Cléon F, Trent MS. 2021. Cardiolipin aids in lipopolysaccharide transport to the gram-negative outer membrane. Proc Natl Acad Sci U S A 118.

10. Madeira F, Park YM, Lee J, Buso N, Gur T, Madhusoodanan N, Basutkar P, Tivey ARN, Potter SC, Finn RD, Lopez R. 2019. The EMBL-EBI search and sequence analysis tools APIs in 2019. Nucleic Acids Res 47:W636–W641.

11. Almagro Armenteros JJ, Tsirigos KD, Sønderby CK, Petersen TN, Winther O, Brunak S, von Heijne G, Nielsen H. 2019. SignalP 5.0 improves signal peptide predictions using deep neural networks. Nat Biotechnol 37:420–423.

12. Mistry J, Chuguransky S, Williams L, Qureshi M, Salazar GA, Sonnhammer ELL, Tosatto SCE, Paladin L, Raj S, Richardson LJ, Finn RD, Bateman A. 2021. Pfam: The protein families database in 2021. Nucleic Acids Res 49:D412–D419.
